# Single-cell gene profiling and lineage tracing analyses revealed novel mechanisms of endothelial repair by progenitors

**DOI:** 10.1007/s00018-020-03480-4

**Published:** 2020-03-13

**Authors:** Jiacheng Deng, Zhichao Ni, Wenduo Gu, Qishan Chen, Witold Norbert Nowak, Ting Chen, Shirin Issa Bhaloo, Zhongyi Zhang, Yanhua Hu, Bin Zhou, Li Zhang, Qingbo Xu

**Affiliations:** 1 Department of Cardiology, The First Affiliated Hospital, School of Medicine, Zhejiang University, 79 Qingchun Road, Hangzhou, 310003 Zhejiang China; 2 School of Cardiovascular Medicine and Science, BHF Centre, King’s College London, 125 Coldharbour Lane, London, SE5 9NU UK; 3 State Key Laboratory of Cell Biology, CAS Center for Excellence in Molecular Cell Science, Institute of Biochemistry and Cell Biology, Shanghai Institutes for Biological Sciences, University of Chinese Academy of Sciences, Chinese Academic of Sciences, Shanghai, 200031 China

**Keywords:** Endothelial repair, Lineage tracing, Metabolism, Single-cell RNA-sequencing, Stem cells

## Abstract

Stem/progenitor cells (SPCs) have been implicated to participate in vascular repair. However, the exact role of SPCs in endothelial repair of large vessels still remains controversial. This study aimed to delineate the cellular heterogeneity and possible functional role of endogenous vascular SPCs in large vessels. Using single-cell RNA-sequencing (scRNA-seq) and genetic lineage tracing mouse models, we uncovered the cellular heterogeneity of SPCs, i.e., c-Kit^+^ cells in the mouse aorta, and found that endogenous c-Kit^+^ cells acquire endothelial cell fate in the aorta under both physiological and pathological conditions. While c-Kit^+^ cells contribute to aortic endothelial turnover in the atheroprone regions during homeostasis, recipient c-Kit^+^ cells of nonbone marrow source replace both luminal and microvessel endothelial cells in transplant arteriosclerosis. Single-cell pseudotime analysis of scRNA-seq data and in vitro cell experiments suggest that vascular SPCs display endothelial differentiation potential and undergo metabolic reprogramming during cell differentiation, in which AKT/mTOR-dependent glycolysis is critical for endothelial gene expression. These findings demonstrate a critical role for c-Kit lineage cells in aortic endothelial turnover and replacement, and may provide insights into therapeutic strategies for vascular diseases.

**Electronic supplementary material:**

The online version of this article (10.1007/s00018-020-03480-4) contains supplementary material, which is available to authorized users.

## Introduction

Previous studies have demonstrated a role of resident stem/progenitor cells (SPCs) in vascular repair and regeneration under both physiological and pathological conditions [1]. Our group previously identified a population of vascular resident SPCs expressing stem cell antigen-1 (Sca-1), c-Kit, and CD34 in the aortic adventitia [2]. Isolated vascular SPCs, including c-Kit^+^ SPCs, display multilineage potential and differentiate into endothelial cells (ECs) and smooth muscle cells (SMCs) to regenerate vascular blood vessels in vivo via cell implantation experiments [2–4]. More recently, vascular SPCs have also been shown to generate macrophages and even cardiomyocytes [5, 6]. These findings suggest a potential therapeutic application of vascular SPCs in regenerative medicine. However, whether endogenous vascular SPCs exhibit multilineage potential or involve in vascular regeneration in vivo still remains controversial.

Recent advances in single-cell RNA-sequencing (scRNA-seq) technologies have allowed for comprehensive analysis of individual cells in organs/tissues [7]. Using scRNA-seq analysis, we have characterized the transcriptomic profile of vascular adventitia and peri-vascular adipose tissue at a single-cell level and determined their role in vascular remodeling through differentiation towards smooth muscle lineage [8, 9]. Substantial heterogeneity and subpopulations of SPCs were observed at the single-cell level in primary peri-vascular tissues [8, 9]. On the other hand, genetic lineage tracing has been proven a useful tool to label endogenous cells and track the cell fate during embryonic development, homeostasis, and in diseased conditions [10]. Recently, several studies using this technique, however, have raised concerns about the actual function and the effectiveness of tissue-resident SPCs in regenerative medicine. Genetic lineage tracing of c-Kit^+^ cardiac stem cells [11–13] or Sca-1^+^ cardiac stem cells [14–17] reveals a minimal role of these cells in cardiomyocyte regeneration. These data challenged the previously defined identity and function of vascular SPCs, and raised the question whether SPCs endogenously regenerate vascular cells like ECs in large vessels. Therefore, the exact identity and the actual function of vascular SPCs in vivo warrant further investigation.

In this study, we first performed scRNA-seq analysis of the whole aorta and uncovered the cellular heterogeneity of vascular SPCs including c-Kit^+^ cells in the aorta in an unbiased manner. To further test the regenerative potential of endogenous c-Kit^+^ cells in vivo, we used an inducible genetic lineage tracing mouse model to label and trace the cell fate of endogenous c-Kit^+^ cells. Results showed that c-Kit^+^ cells are a major source of endothelial cells in atheroprone regions of aorta, and transplant arteriosclerotic lesions. Further pseudotime trajectory analysis of scRNA-seq data and in vitro cell experiments validated the endothelial differentiation potential of vascular c-Kit^+^ SPCs via an AKT/mTOR-dependent glycolysis axis.

## Materials and methods

### Animal experiments

All animal experiments and protocols were approved by the Institutional Committee for the Use and Care of Laboratory Animals and the UK Home Office (PPL70/8944S). C57BL/6J and BALB/c mice were purchased from Harlan, except that mice used for scRNA-seq (12-week-old male wild-type C57BL/6J and ApoE–/– mice) were purchased from the Jackson Laboratory. Rosa26-tdTomato mice [18] (B6.Cg-Gt(ROSA)26Sor^tm9(CAG−tdTomato)Hze^/J, 007909) were also purchased from the Jackson Laboratory. Kit-CreER mice (C57BL/6 background) were kindly provided by Bin Zhou [19]. We crossed Kit-CreER mice with Rosa26-tdTomato mice to generate Kit-CreER; Rosa26-tdTomato mice. Tamoxifen (Sigma, T5648, 0.1–0.15 mg/g body weight) dissolved in corn oil (Sigma, C8267, 20 mg/mL) were intraperitoneally administered to Kit-CreER; Rosa26-tdTomato mice to activate tdTomato labeling. Mice were maintained at 22 °C under a 12-h light and 12-h dark cycle and fed with chow diet. Both male and female mice were used in our experiments and were randomly allocated to different experimental groups, except that only male mice were used for scRNA-seq analysis to avoid data variation caused by sex difference [20].

Allograft transplantation was performed as previously described [21]. In brief, donor aortic segments were harvested and then implanted into the carotid artery of recipient mice. A Nikon stereo microscope (SMZ1270) was used to perform whole mount bright field and fluorescence analysis of donor aortas. Allograft transplantation was performed between BALB/c (H2^d^) mice and mice on C57BL/6J (H2^b^) background (including Kit-CreER; Rosa26-tdTomato mice and chimeric mice described below). Bone marrow transplantation was performed to create chimeric mice as previously described [22], between donor Kit-CreER; Rosa26-tdTomato mice and recipient C57BL/6J mice. Briefly, bone marrow cells were harvested from the donor mice, passed through 40-μm cell strainers (Falcon, 352340) and then resuspended in serum-free DMEM (ATCC, 30-2002) before cell transplantation. Recipient mice first received a lethal dose of whole-body irradiation (900 rads) and further received 5 × 10^6^ donor bone marrow cells via tail vein injection 6 h later. The investigators harvesting the tissues and collecting the data were blinded to the groups.

### Isolation of single cells

For isolation of single cells from the whole aorta of 12-week-old male wild-type and ApoE–/– mice, a similar protocol was followed as previously described [8]. A detailed method is available in the detailed methods of the Supplement. Isolated single cells were stained with LIVE/DEAD ™ Fixable Near-IR Dead Cell Stain Kit (Invitrogen, L34975, 1:1000) and Hoechst 33342 (Invitrogen, H3570, 1:1000) for 20 min. Specifically, digested cells from the medial/intimal layers were first stained with CD31-AF647 (Biolegend, 102516, 1:1000) for an additional 20 min. After PBS washing, cells were resuspended in PBS and single nucleated live cells (Hoechst^+^/Dead Cell Stain^−^) were sorted into PBS with 0.04% BSA using a BD FACS ARIA II Flow Cytometer (BD Biosciences). For cells from the medial/intimal layers, endothelial cells (CD31^+^Hoechst^+^/Dead Cell Stain^−^) and non-endothelial cells (CD31^−^Hoechst^+^/Dead Cell Stain^−^) were sorted into PBS with 0.04% BSA. A single-cell preparation comprising approximately 50% endothelial cells and 50% non-endothelial cells (mostly smooth muscle cells) was then prepared. For isolation of single tdTomato^+^ cells from the aortic grafts, a similar protocol was used as describe above. Specifically, aortic grafts from 14- or 16-week-old Kit-CreER; Rosa26-tdTomato mice were isolated for scRNA-seq analysis. Following a similar digestion protocol as described above, all digested cells from one sample were pooled together at the end of the digestion process. Digested cells were then stained with DRAQ5 (Abcam, ab108410, 1:1000) and CD31-FITC (Invitrogen, 11-0311-81, 1:1000) for 30 min. DAPI was added to the cell just before loading to flow cytometer. Single nucleated live tdTomato^+^ cells (DRAQ^+^/DAPI^−^) were sorted into PBS with 0.04% BSA using a BD FACS ARIA II Flow Cytometer (BD Biosciences).

### Single-cell RNA-sequencing and data analysis

For single-cell RNA-sequencing (scRNA-seq) of four pooled samples obtained from the adventitia and the intimal/medial layers of aorta, from wild-type or ApoE–/– mice, a Chromium™ Single Cell 3′ Reagent Kit v2 chemistry (10× Genomics) was used and a standard protocol was followed. Library was generated and sequenced on a NovaSeq platform (Illumina) with paired-end 150 bp (PE 150) sequencing strategy. Single-cell RNA-sequencing raw data were processed using Cell Ranger (version 2.1.1), resulting in the four data sets of adventitial and intimal/medial layers of aorta from wild-type and ApoE–/– mice. It should be noted that, the data sets of aortic adventitia part have been recently used and published to depict the adventitial cell atlas in mouse aorta [8]. However, c-Kit^+^ cells have not been assessed in the paper. The role of c-Kit^+^ cells in the scRNA-seq of whole aorta has also not been determined yet. Here, we further aggregated the data sets from both the adventitial and intimal/medial layers of aorta using “cellranger aggr”, and assessed c-Kit^+^ cells in the whole aorta. After aggregation of the samples, Seurat [23] v3 was used for downstream analyses, including cell clustering and identification of differentially expressed genes. Integrating analysis of the whole aorta data from our data set, with three other published scRNA-seq data sets [24–27], was also analyzed using Seurat v3. A standard integrated analysis was performed with default parameters.

For scRNA-seq of tdTomato^+^ cells obtained from aortic grafts, a similar method was used as described above. Specifically, a Chromium™ Single Cell 3′ Reagent Kit v3 chemistry (10× Genomics) was used and a standard protocol was followed. Library was generated and sequenced on a Hiseq 2500 System (Illumina). Single-cell RNA-sequencing raw data were processed using Cell Ranger (version 3.0.2). Aggregation data were also analyzed by Seurat v3 for cell clustering and differentially expressed genes following a standard protocol. Pseudotime trajectory analysis was performed using Monocle 2 [28]. We used differentially expressed genes acquired from the Seurat analysis to order cells in pseudotime. For analysis of PI3K-AKT-mTOR signaling over pseudotime, we used a gene set “HALLMARK_PI3K_AKT_MTOR_SIGNALING” from GSEA, which includes genes up-regulated by activation of the PI3K/AKT/mTOR pathway. Human gene names were first converted to mouse gene names using “biomaRt” [29] before analysis. Gene set enrichment analysis of KEGG pathway was analyzed using DAVID [30] bioinformatics resources. Detailed methods to analyze scRNA-seq data sets are available in the supplementary methods.

### Immunofluorescence staining

For immunofluorescence staining of cryo-sections from mouse tissues, tissues were first collected, washed with PBS, and fixed in 4% paraformaldehyde (PFA, Santa Cruz, sc-281692) at 4 °C for 2 h, followed by dehydration in 30% sucrose solution (BDH AnalaR, 102747E) at 4 °C overnight. Tissues were then embedded in OCT, frozen at − 80 °C for storage, or cut into 10-µm sections by a CryoStar Cryostat (Thermo Scientific). For en face staining of aorta, the whole aorta was first collected from mice and the adventitial layer was carefully removed. Aorta was then cut open longitudinally with the endothelial layer facing up and fixed in 4% PFA. For immunostaining of cultured cells, cells were washed with PBS and then fixed in 4% PFA for 15 min. All the aforementioned samples were then blocked and permeabilized in 5% donkey serum (with 0.1% Triton X-100) for 1 h, stained with primary antibodies overnight, and then incubated with Alexa Fluor-conjugated secondary antibodies (Invitrogen, 1:500) for 1 h, followed by DAPI (Molecular Probe, D1306) staining and mounting in anti-fade mounting medium (Dako, s3023). Primary antibodies used in this study included c-Kit (R&D, AF1356, 1:50), Sca-1 (Abcam, ab51317, 1:200), CD34 (BD Pharmingen, 553731, 1:100), CD45 (Abcam, ab10558, 1:100), tdTomato (Rockland, 600-401-379, 1:500), CD144 (Santa Cruz, sc-6458, 1:100), CD144 (R&D, AF1002, 1:50), CD31 (BD Pharmingen, 553370, 1:100), VCAM1 (Santa Cruz, sc-53462,1:200), and e-NOS (BD Pharmingen, 610297, 1:200). A Leica (TCS SP5) confocal microscope was used to acquire images, further analyzed by Leica Application Suite AF Software. A secondary antibody only control was used in immunofluorescence staining.

### Flow cytometry

For staining of cells from bone marrow and blood, cells were first harvested and incubated with red blood cell lysis buffer (eBioscience, 00-4333) to lyse red blood cells, and passed through a 40-μm cell strainer to obtain single-cell suspensions. Cells were then stained with DAPI (Molecular Probe, D1306) to exclude dead cells from the analysis where indicated, and further analyzed for tdTomato expression. For staining of vascular c-Kit^+^ SPCs, cells were first stained with indicated conjugated-antibodies (1:50) for 30 min on ice. Conjugated-antibodies used in this study include c-Kit-PE (Biolegend, 105807), Sca-1-PE (BD biosciences, 553108), CD31-PE/Cy7 (Biolegend, 102523), CD34-AF647 (Biolegend, 103124), CD29-FITC (Abcam, ab21845), CD45-PerCP/Cy5.5 (Biolegend, 103131), CD105-PE (Biolegend, 120407), CD140a-PE (eBioscience, 12-1401-81), and α-SMA-AF488 (Abcam, ab184675). Corresponding control isotype antibodies were used as control. For analyses of glucose uptake, 2-NBDG (100 μM, Invitrogen, N13195) was added to cultured cells and incubated for 1 h at 37 °C. Cells were then washed with PBS, detached by trypsin, and suspended in PBS for further analysis. All prepared samples were analyzed by a BD LSR Fortessa II flow cytometer (Becton Dickinson) or BD ACCURI C6 flow cytometer (BD Biosciences). FlowJo v10 software (Tree Star) was used to analyze the flow cytometric data.

### Mouse vascular adventitial c-Kit^+^ SPC isolation, cell culture, and differentiation

Mouse vascular SPCs were isolated as previously described [2]. Briefly, the whole aorta was harvested; aortic adventitial layers were carefully dissected from the medial and intimal layers. The isolated aortic adventitia was then cut into small pieces, seeded in a 0.04% gelatin (Sigma, G1393)-coated T25 flask, and maintained in complete stem cell culture medium, which consists of DMEM (ATCC, 30-2002), 10% EmbryoMax ES Cell Qualified FBS (Millipore, ES-009-B), 10-ng/mL leukemia inhibitory factor (Merck Millipore, LIF1050), 0.1-mM 2-mercaptoethanol (Gibco, 31350–010), 100 U/mL penicillin–streptomycin (Gibco, 15140122), and 2-mM L-glutamine (Gibco, 25030081). Cells derived from the outgrowth of aortic adventitia were then passaged for cell enrichment. c-Kit^+^ cells were then sorted from these enriched cells using c-Kit (Miltenyi Biotec, 130-091-224) microbeads according to the manufacturer’s instructions. Isolated c-Kit^+^ SPCs were maintained in complete stem cell culture medium for further cell culture. c-Kit^+^ SPCs between passages 5–15 were used in our cell experiments. For induction of endothelial cell differentiation, c-Kit^+^ SPCs were maintained in EGM™-2 endothelial cell growth medium-2 (Lonza, CC-3162). Cells were treated with indicated doses of chemical inhibitors including 2-deoxy-d-glucose (2DG, Sigma, D8375) and Rapamycin (Selleck Chemicals, S1039) where indicated.

### Western blot analysis

Proteins were extracted by lysing cells in RIPA buffer (Thermo Fisher, 89901) supplemented with phosphatase inhibitor tablets (Roche, 04906837001) and protease inhibitor tablet (Roche, 05892970001). Samples mixed with LDS sample buffer (Invitrogen, NP0007) were loaded on NuPAGE 4–12% Bis Tris-gels (Thermo Fisher, NP0321) for protein separation, transferred to nitrocellulose membranes (GE healthcare, RPN303LFP), and blocked in 5% milk or BSA for 1 h at room temperature. Membranes were then incubated with primary antibody at 4 °C overnight and conjugated with IRDye secondary antibody (LI-COR) for 1 h at room temperature. Membranes were detected by Odyssey CLx near-infrared fluorescence imaging system and analyzed by Image Studio software (LI-COR Biosciences). Primary antibodies used in this study include CD31 (Abcam, ab28364, 1:1000), CD144 (Abcam, ab33168, 1:1000), GAPDH (Abcam, ab9485, 1:1000), GAPDH (Abcam, ab8245, 1:1000), HK1 (Cell Signaling, 2024S, 1:1000), PDH (Cell Signaling, 3205S, 1:1000), HK2 (Cell Signaling, 2867S, 1:1000), total OXPHOS rodent WB antibody cocktail which contains antibodies including mitochondrial complex I–V (Abcam, ab110413, 1:1000), p-mTOR (Cell Signaling, 5536S, 1:1000), p-AKT (Cell Signaling, 4060S, 1:1000), AKT (Cell Signaling, 2920S, 1:1000), and p-4EBP1 (Cell Signaling, 2855S, 1:1000).

### RNA extraction, reverse transcription, and quantitative polymerase chain reaction

RNA extraction was performed via using an RNeasy Mini Kit (Qiagen, 74106). QuantiTect Reverse Transcription Kit (Qiagen, 205311) was used for reverse transcription of mRNA to cDNA and further amplification. Quantitative polymerase chain reaction was performed using SyGreen Blue Mix Hi-ROX (PCR Biosystems, PB20.16-51). All experiments were performed according to the manufacturer’s instructions. Primer sequences used in this study are listed in Table IV of the online-only Data Supplement.

### Measurement of acetylated low-density lipoprotein uptake

To measure acetylated low-density lipoprotein (ac-LDL) uptake, live cells were incubated with 10 μg/mL Alexa Fluor 594 conjugated Ac-LDL (ThermoFisher, L-35353) for 4 h and further stained with Hoechst 33342 (Invitrogen, H3570). Images were acquired by an Olympus IX-81 microscope and analyzed by Image J software.

### Extracellular flux analysis

Seahorse XFe24 Analyzer (Seahorse Bioscience) was used to measure OCR and ECAR. Cells (30,000 cells per well) were seeded in a gelatin-coated XF24 cell culture microplate. Glycolysis stress tests were performed to monitor ECAR under basal conditions and sequential stress conditions in response to 10-mM glucose (Sigma, G7021), 1-μM oligomycin (Sigma, 75351), and 100-mM 2DG. Mitochondrial stress tests were performed to monitor OCR and ECAR under basal conditions and sequential stress conditions in response to 1-μM oligomycin, 1-μM carbonyl cyanide 4-trifluoromethoxy phenylhydrazone (FCCP, Sigma, C2920), and 1-μM rotenone (Sigma, R8875) plus 1-μM antimycin (Sigma, A8674). Cell numbers were counted for data normalization after metabolic assays. Analyses of OCR and ECAR in all metabolic stress tests were performed using Wave software (Seahorse Bioscience).

### Luciferase activity assay

Cells were seeded in gelatin-coated 24-well plates and cultured in indicated conditions. After 48 h, cells were transfected with a pGL3-Luc-CD144 promoter reporter vector (0.5 μg/well) using Lipofectamine™ 3000 Transfection Reagent (Invitrogen, L3000015) following the manufacturer’s protocol. pGL3-Luc-Renilla (0.1 μg/well) was also included in all transfections as an internal control. The firefly and renilla luciferase activities were detected using Dual-Luciferase Reporter Assay System (Promega, E1910) 48 h after transfection, and measured by the Tecan Infinite 200 PRO. Relative luciferase unit was defined as the ratio of firefly luciferase activity of specific gene to renilla luciferase activity with control set as 1.

### Data availability

The data that support the findings of this study are available on reasonable request. ScRNA-seq data of our study are available in Gene Expression Omnibus (GEO) repository (GSE140811). Two previously published scRNA-seq data sets used in this study are also available in the GEO repository: GSE117963 [24] (Dataset 2); GSE109774 [26] (Dataset 4, the scRNA-seq data of aorta (FACS) in the Tabula Muris project, https://tabula-muris.ds.czbiohub.org/). Dataset 3 [27] is available via the Broad Institute Single Cell Portal (https://portals.broadinstitute.org/single_cell/study/SCP289/single-cell-analysis-of-the-normal-mouse-aorta-reveals-functionally-distinct-endothelial-cell-populations).

### Statistical analyses

All data are presented as mean and SEM. GraphPad Prism 7 was used to perform statistical analysis. Data were first analyzed for normality test, followed by unpaired Student’s t test, or one-way ANOVA with Dunnett’s or Tukey’s post hoc tests where indicated in the figure legends. *P* < 0.05 was considered to be statistically significant.

## Results

### Single-cell transcriptome analyses of vascular SPC in the mouse aorta

**Fig. 1:**
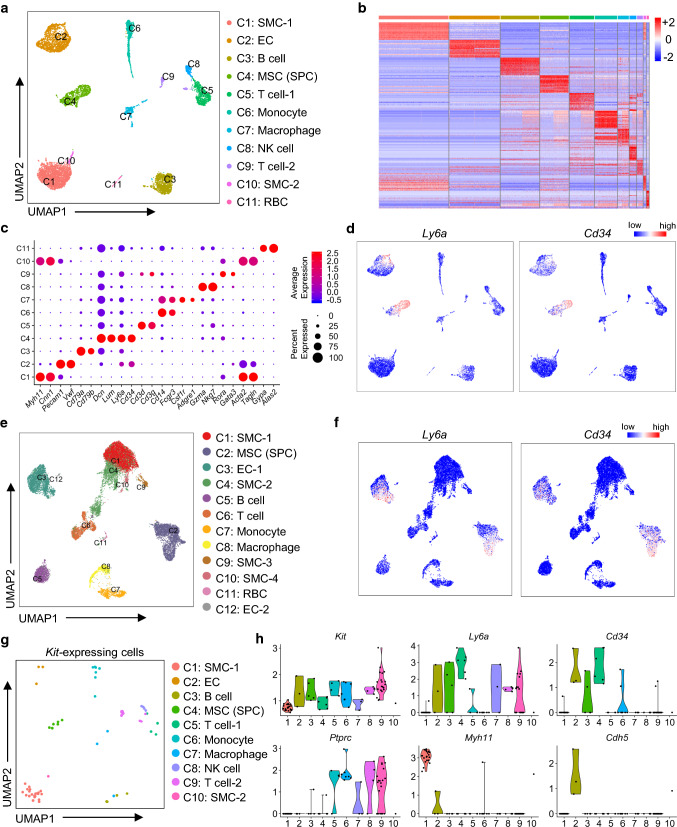
Single-cell RNA-sequencing analysis reveals cellular heterogeneity of SPC, including *Kit*-expressing cells in the whole aorta. **a** UMAP plot showing 11 color-coded cell clusters identified using aggregation data of wild-type and ApoE–/– mice. *n* = 9745 individual cells. Putative biological identity of each cell cluster is defined on the right. **b** Heatmap showing top 20 differentially expressed genes in each cell cluster. Normalized gene expression is shown. **c** Dot plot showing average expression (color-scaled) of selected cell marker genes in each cell cluster. Dot size reflects the proportion of cells expressing the selected gene. **d** Feature plots showing average expression (color-scaled) of stem progenitor markers *Ly6a* and *Cd34* in each cell cluster. **e** UMAP plot showing 12 color-coded cell clusters from integrated analysis of four scRNA-seq data sets. *n* = 19,234 individual cells. Putative biological identity of each cell cluster is defined on the right. **f** Feature plots showing average expression (color-scaled) of stem progenitor markers *Ly6a* and *Cd34* in each cell cluster of integrated data. **g** UMAP plot showing *Kit*-expressing cells in each cell cluster of **a**. Cell clusters are re-color-coded and putative biological identity of each cell cluster is re-defined on the right. *n* = 75 individual cells. **h** Violin plots showing normalized expression levels of *Kit*, *Ly6a*, *Cd34*, *Ptprc*, *Myh11,* and *Cdh5* in each cell cluster of **a**. Only *Kit*-expressing cells are shown in these violin plots

Previous studies have suggested a multilineage potential and cellular heterogeneity of vascular SPCs. To clearly delineate the heterogeneity of SPC (including c-Kit^+^ cells) in the mouse aorta, we first performed scRNA-seq (10 × Chromium) on cells isolated from the whole aorta from both wild-type and ApoE–/– mice. To identify as many cell types as possible, single nucleated live cells from the aortic adventitia and the intimal/medial layers were separately digested, sorted, and further subjected to scRNA-seq (Fig. S1a). Specifically, to avoid oversampling of SMCs, the major cell population in the intimal/medial layers, CD31^+^ ECs and CD31^−^ non-ECs were sorted and mixed at a 1:1 ratio before scRNA-seq (Fig. S1a, b). scRNA-seq data from the adventitia and intimal/medial layers were then aggregated for further analyses (Fig. S2a). In our data set, approximately 9745 cells passed quality control metrics and were further analyzed (Fig. S2b–f). Unsupervised clustering using Seurat [23] identified 11 cell clusters, which were further attributed to putative biological identities based on the expression of differentially expressed genes (Fig. 1a–c; Table S1), including SMC (2 clusters; *Myh11*, *Cnn1*, *Acta2*, *Tagln*), EC (*Pecam1*, *Vwf*), B cell (*Cd79a*, *Cd79b*), mesenchymal stromal cell (MSC, *Lum, Dcn*), T cell (2 clusters; *Cd3d*, *Cd3g, Rora, Gata3*), monocyte (*Cd14*, *Fcgr3*), monocyte/macrophage (*Cd14*, *Fcgr3*, *Csf1r*, *Adgre1*), natural killer (NK) cells (*Gzma*, *Nkg7*), and red blood cells (RBC, *Gypa*, *Alas2*).

We next analyzed the expression of previously reported vascular stem/progenitor markers [1, 31] in our data set. Interestingly, high levels of two classical progenitor markers, *Ly6a* and *Cd34*, were detected in a large population of MSC, as well as some populations of EC (Fig. 1d). To further confirm the distribution of vascular SPCs, another 21 vascular stem/progenitor markers were also assessed (Fig. S3). Consistently, high levels of *Pdgfra*, *Pdgfrb*, *Itgb1*, *Cd44*, *Eng*, *Thy1,* and *Procr* were detected in a large population of *Ly6a*^hi^ and *Cd34*^hi^ MSC, while some expression of *Kit*, *Kdr*, *Gli1*, *Nefm*, *Nes*, *Bst1*, *Emcn*, *Mcam*, *Cspg4, Nt5e, *and *Ptprc* were also observed. It should be noted that many of these progenitor markers were detected in different cell populations. Although these data undoubtedly support the presence of vascular SPC populations, the expression of vascular stem cell markers in heterogeneous cell populations may suggest that different cell populations may also harbor its own stem cell subpopulation, or that some markers can also be expressed in certain mature cell populations. To further confirm the validity of our data, we also compared our dataset with three recent published scRNA-seq analyses of mouse aorta [24, 26, 27]. Integrated analyses of four data sets identified 12 cell clusters, with similar biological identities as our single data set (Figs. 1e; S4a–c, Table S2). A population of SPCs expressed high levels of *Ly6a*, *Cd34* (Fig. 1f), as well as many other vascular stem cell markers including c-Kit (Fig. S4d). Taken together, scRNA-seq analyses identified a heterogeneous cell population expressing vascular stem cell markers, including a population of c-Kit^+^ cells in SPC population.

Here, we further focused on a specific population of vascular SPCs, c-Kit^+^ cells, a population that has been controversially debated recently. From both individual and integrated analyses, c-Kit^+^ was observed to be expressed in a minor population of cells in the aorta (Figs. S3b and 4d). To gain a better understanding of vascular c-Kit^+^ cells, *Kit*^+^ cells were extracted from our data set for further analyses (Figs. 1g; S5a). Of the total 9745 cells, only 75 cells showed the expression of *Kit*, suggesting that *Kit*-expressing cells are a minor population in the mouse aorta. Interestingly, *Kit* was detected in ten of the 11 identified cell clusters, with most cell clusters showing a low proportion of *Kit*^+^ cells (Fig. S5b). Similar results were obtained in the integrated data sets (Fig. S5c). Specifically, *Kit* was detected in the adventitial SPC population, which showed the highest co-expression of *Ly6a* and *Cd34*, as well as several mesenchymal markers (Fig. 1h; Table S3). *Kit* expression was also observed in the cell clusters of SMC (~ 1%) and EC (~ 0.2%), suggesting that c-Kit^+^ cells may represent a rare population of these vascular cells in the large vessels. Besides, immune cell clusters (*Ptprc*^+^ cells) also express *Kit*. We suppose that these immune populations may be mature immune cells, as DC and NK cells retain c-Kit expression even upon cell differentiation [32], or may be a population of previously identified vascular progenitors of immune cells [5]. Collectively, these data suggest that c-Kit^+^ cells are minor and heterogeneous cell populations in the mouse aorta, consisting of adventitial SPCs, a rare population of SMCs and ECs, mature or vascular progenitors of immune cells.

### Lineage tracing study reveals the distribution of c-Kit^+^ cells in the mouse aorta

**Fig. 2:**
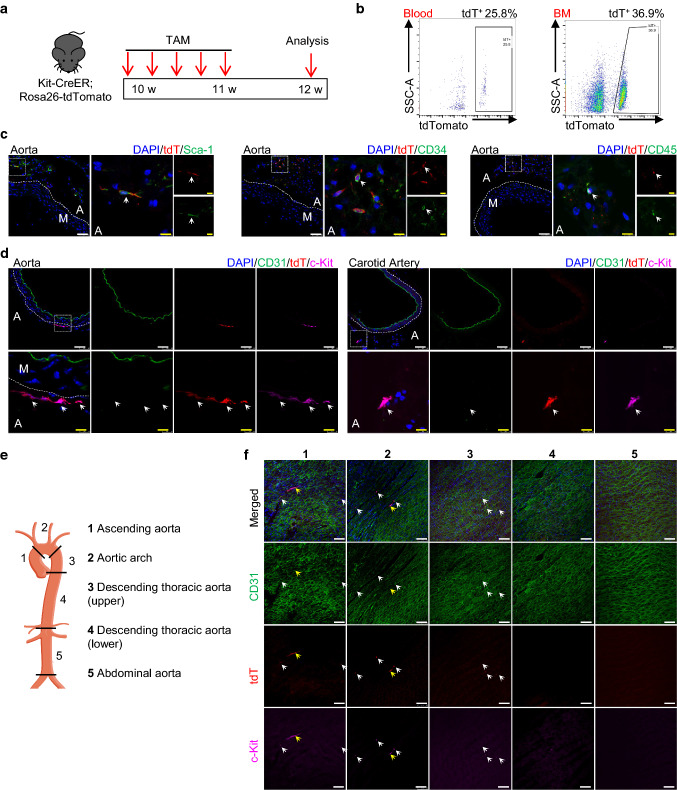
Lineage tracing showing the distribution of tdTomato-labelled c-Kit^+^ cells in the blood vessels. **a** Schematic showing tamoxifen-induced tdTomato labelling of c-Kit^+^ cells using Kit-CreER; Rosa26-tdTomato mice (*n* = 6). Mice were treated with tamoxifen for 1 week, and aortas were harvested and analyzed 1 week later. **b** Representative flow cytometric analysis of tdTomato^+^ cells in the blood and bone marrow. **c** Representative images showing staining of tdTomato, Sca-1, CD34, and CD45 in tamoxifen-treated mice. Arrows indicate examples of co-staining cells. **d** Representative immunostaining of cross sections showing the expression of CD31, tdTomato, and c-Kit in the aorta and carotid artery. Arrows indicate examples of tdT^+^c-Kit^+^ cells. **e** Aorta was divided into five segments for further analysis and numbered as follows: 1, ascending aorta; 2, aortic arch; 3, descending thoracic aorta (upper); 4, descending thoracic aorta (lower); 5, abdominal aorta. **f** Representative en face staining of aortic intimal layers from different segments showing expression of tdTomato, CD31, and c-Kit. Arrows indicate examples of tdT^+^c-Kit^+^ cells. Scale bars, 50 μm (white) and 10 μm (yellow). TAM, tamoxifen; BM, bone marrow; A, adventitia; M, media; tdT, tdTomato

To investigate the endogenous protein expression of c-Kit and endothelial differentiation of c-Kit^+^ cells in the mouse aorta, we used an inducible genetic lineage tracing mouse model, Kit-CreER; Rosa26-tdTomato mice. Mice were treated with five consecutive pulses of tamoxifen for 1 week to induce tdTomato labeling of c-Kit^+^ cells, and tissues were collected for analysis 1 week later (Fig. 2a). This inducible lineage tracing model is suitable for tracing the cell fate of c-Kit^+^ cells, as it allows for permanent tdTomato labeling of c-Kit^+^ cells even if the cells lose the expression of c-Kit protein. Flow cytometric results showed the successful labeling of tdTomato^+^ cells in the blood and bone marrow, compared to control group (Figs. 2b and S6a–b). Moreover, co-expression of c-Kit and tdTomato was widely observed in the lung and spleen (Fig. S6c), which are known c-Kit-expressing organs, confirming the high labeling efficiency of c-Kit in this mouse model. We next focused on the expression of c-Kit in the blood vessels. Consistent with our scRNA-seq data, tdTomato were found to be expressed in adventitial Sca-1^+^ cells, CD34^+^ cells, and CD45^+^ cells (Fig. 2c), suggesting that c-Kit labels a heterogeneous population of adventitial SPCs, mature or progenitors of immune cells. Of interest, tdTomato-labeled c-Kit^+^ cells were mainly found in the aortic adventitia, but not CD31^+^ endothelial layer in the cryo-sections (Fig. 2d). Similar results were observed in the carotid artery.

We performed en face staining of the whole aorta, which allows us to analyze all cells on the intimal layers of the vessel wall. The whole aorta was divided into five regions (including the ascending aorta, the aortic arch, the upper and lower parts of the descending thoracic aorta, and the abdominal aorta) (Fig. 2e). En face analysis of the intimal layers showed that only a very few tdTomato-labeled c-Kit^+^ cells were observed across the whole aorta (Fig. 2f). These tdTomato^+^ cells were mainly in the atheroprone regions (regions 1–3, ascending aorta, aortic arch, and upper curved part of descending thoracic aorta), but not athero-resistant regions of aorta. Most of the observed tdTomato-labeled cells showed a round and dotted shape and were much smaller than CD31^+^ ECs (Fig. 2f, white arrows), whereas some tdTomato-labeled cells showed a bigger and elongated shape (Fig. 2f, yellow arrows). We supposed that these small dotted cells could be immune cells attached to the intimal layer, while the bigger cells might be tdTomato-labeled c-Kit^+^ ECs. Based on these observations, it is likely that c-Kit endogenously labels a rare population of intimal ECs in the aorta. Taken together, these results indicate that c-Kit labels a heterogeneous population, including a population of adventitial SPCs, mature or progenitors of immune cells, and a possible rare population of intimal ECs, in the mouse aorta.

### c-Kit^+^ cells adopt endothelial cell fate in atheroprone regions of aorta under homeostasis

**Fig. 3:**
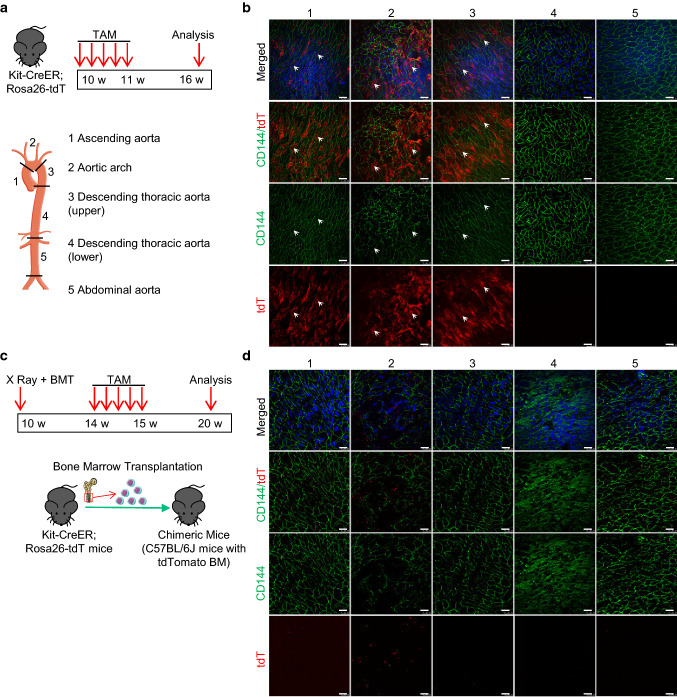
Nonbone marrow c-Kit^+^ cells are involved in endothelial turnover in the atheroprone regions of mouse aorta. **a** Kit-CreER; Rosa26-tdTomato mice were pulsed with tamoxifen for 1 week and analyzed 5 weeks later. Aorta was then divided into five segments for further analysis (*n* = 6). **b** Representative en face staining of aortic intimal layers showing expression of tdTomato, CD144 in different aortic segments in Kit-CreER; Rosa26-tdTomato mice as described in **a**. Arrows indicate tdTomato-labeled CD144^+^ ECs. **c** Strategy for creation of chimeric mouse model. Irradiated C57BL/6 J mice were transplanted with bone marrow cells from Kit-CreER; Rosa26-tdTomato mice, and then treated with tamoxifen and harvested for further analysis (*n* = 6). **d** Representative en face staining of aortic intimal layers showing expression of tdTomato, CD144 in chimeric mice. Scale bars, 25 μm. TAM, tamoxifen; BM(T), bone marrow (transplantation); tdT, tdTomato

Our previous study has demonstrated a high endothelial turnover rate in the atheroprone regions of aorta, in which SPCs may play a role [33]. The evidence that a rare population of aortic c-Kit^+^ ECs in the atheroprone regions, together with the previously defined c-Kit^+^ SPCs in the aortic adventitia were endogenously detected, may suggest a possible role of c-Kit^+^ cells in this process. To test this hypothesis, the same tamoxifen induction strategy was applied, and we collected tissues for analysis five weeks later (Fig. 3a). Successful labeling of tdTomato^+^ cells was confirmed in the bone marrow by flow cytometric analysis (Fig. S7a). Notably, the percentage of tdTomato-labeled bone marrow cells was much higher in this long-term model, compared to the above short-term mouse model (Fig. S6b). As we used the same tamoxifen induction strategy as the short-term model, which only allows cell labeling of tdTomato for the first week, this suggests a significant cell proliferation of c-Kit^+^ hematopoietic stem/progenitor cells in the bone marrow during homeostasis. The whole aorta was divided into five regions for en face analysis of the aortic intimal layers. Surprisingly, a large number of tdTomato-labeled CD144^+^ ECs were observed in the atheroprone regions (regions 1–3), but again not in the athero-resistant regions (regions 4–5) of the aorta (Fig. 3b). Compared to the above short-term model (Fig. 2f), we observed a significant increase of tdTomato^+^CD144^+^ cells in the atheroprone regions of aorta (Fig. 3b). As only a very few tdTomato^+^CD144^+^ ECs were detected in the short-term model, these data collectively suggest that c-Kit^+^ cells may represent a population of endothelial progenitors and are involved in the endothelial turnover in the atheroprone regions during homeostasis.

Endothelial turnover in the atheroprone sites may require stem/progenitor cell repair, while bone marrow or nonbone marrow tissues can be possible sources of endothelial progenitors [33]. To investigate the origin of these c-Kit-derived ECs, we created a chimeric mouse model, in which wild-type C57BL/6J mice were transplanted with bone marrow cells from Kit-CreER; Rosa26-tdTomato mice (Fig. 3c). These chimeric mice were then pulsed with tamoxifen and analyzed 5 weeks later. Only bone marrow-derived c-Kit^+^ cells are labeled with tdTomato in this model. Flow cytometric analysis showed the successful reconstitution of tdTomato^+^ bone marrow cells (Fig. S7b). Interestingly, en face staining only showed the presence of small round patterns of tdTomato expression in the aortic intimal layers in this chimeric model (Fig. 3d). Consistent with the above data, these tdTomato^+^ cells did not co-express EC marker CD144, but seemingly resided proximal to or on the surface of the endothelial layer. Compared to the athero-resistant regions, the number of these bone marrow-derived small round tdTomato^+^ cells was much higher in the aortic arch (Fig. 3d), which suggests an increased infiltration of bone marrow-derived c-Kit^+^ cells, possibly immune cells, in the atheroprone region. While both bone marrow and nonbone marrow c-Kit^+^ cells are increased in the aorta, these data collectively demonstrate that nonbone marrow c-Kit^+^ cells serve as endothelial progenitors and regenerate aortic ECs in the atheroprone regions during homeostasis.

### c-Kit^+^ cells replace luminal and microvessel ECs in transplant arteriosclerosis

**Fig. 4:**
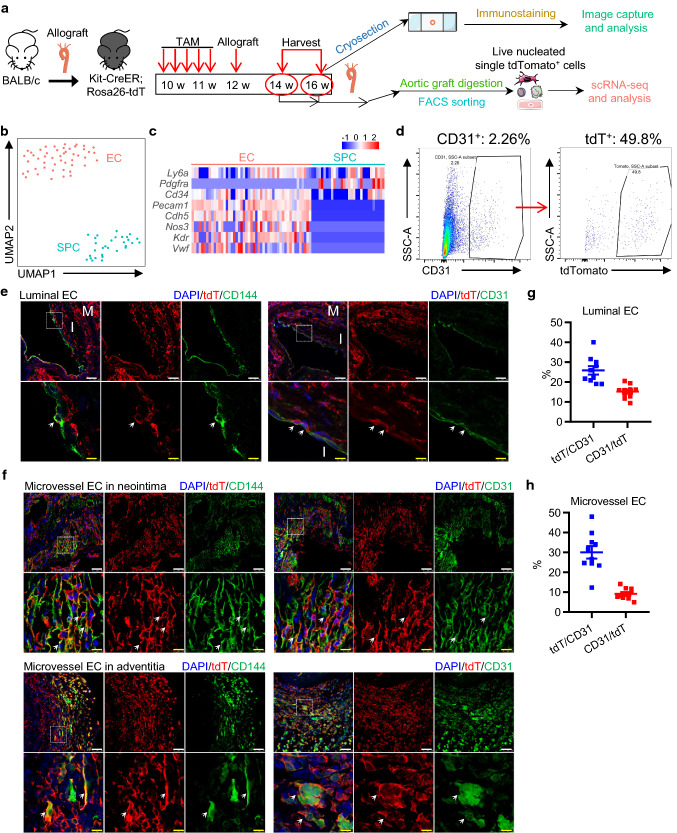
Recipient c-Kit^+^ cells differentiate into ECs in transplant arteriosclerosis. **a** Schematic showing allograft transplantation in Kit-CreER; Rosa26-tdTomato mice. Mice treated with tamoxifen were transplanted with aortic segments from BALB/c mice, followed by graft harvesting two or four weeks after surgery. Aortic grafts were subjected to cryosection (*n* = 6) or scRNA-seq analysis (*n* = 6).** b** UMAP plot showing color-coded nonimmune cell clusters from scRNA-seq analysis of tdTomato^+^ cells pooled from aortic grafts. *n* = 79 cells. **c** Heatmap showing expression of selected EC and SPC marker genes in each cell cluster. **d** Representative flow cytometric analysis showing the percentage of CD31^+^ cells in total cells pooled from the aortic grafts, and the percentage of tdTomato^+^ cells in total CD31^+^ cells. **e** Representative images showing ECs on the luminal surface of 4-week aortic allografts, stained with tdTomato, CD31, and CD144. **f** Representative images showing microvessels in the neointimal region and the adventitial region of 4-week allograft, stained with tdTomato, CD31, and CD144. **g** and **h** Graph showing percentage of tdTomato expression in CD31^+^ ECs and CD31 expression in tdTomato^+^ cells in luminal EC (**g**) or microvessel EC (**h**). Scale bars, 50 μm (white) and 10 μm (yellow). Arrows indicates tdT-labelled ECs. Data shown are mean ± SEM. *n* = 10 per group. TAM, tamoxifen; M, media; I, neointima; tdT, tdTomato

We next evaluated whether c-Kit^+^ cells give rise to endothelial cells under diseased conditions. Previous lineage tracing study has shown a minimal role of c-Kit^+^ cells in EC generation in femoral artery injury and carotid artery ligation models, both of which are mild vessel injury models [34]. Interestingly, a recent study revealed that vascular SPCs participate in SMC replacement after severe vessel injury, but not in mild injury conditions [35]. We have also recently suggested a role of c-Kit^+^ cells in SMC replacement in a mouse model of transplant arteriosclerosis [36]. However, it still remains unclear whether c-Kit^+^ cells give rise to ECs in this model. Here, we further re-used this severe vessel injury mouse model as previously described [36] and assessed the role of c-Kit^+^ cells in EC replacement. Major histocompatibility complex (MHC)-mismatched vascular allo-transplantation was performed between mice on BALB/c (H2^d^) and C57BL/6 (H2^b^) background. This model is a severe vascular injury model caused by allo-immune reactions and may induce cell apoptosis of all vascular layers [37–39]. Kit-CreER; Rosa26-tdTomato mice (C57BL/6J background) were pulsed with tamoxifen, subjected to aortic segment transplantation from BALB/c mice and were sacrificed 2 or 4 weeks after surgery (Fig. 4a). The aortic grafts were then prepared for both cryo-sections and scRNA-seq analysis. Specifically, we sorted tdTomato^+^ cells, the c-Kit-derived cells from the aortic grafts, for scRNA-seq analysis (Fig. S8a). After data aggregation, filtering and unbiased clustering analysis, we identified a mixed population of EC and SPC (Fig. S8b-c), which was further separated to distinct populations of EC (expressing *Pecam1*, *Cdh5*, *Nos3*, *Kdr*, and *Vwf*) and SPC (expressing *Ly6a*, *Cd34*, and *Pdgfra*) by focused clustering analysis (Fig. 4b, c). Given that all cells subjected to scRNA-seq were tdTomato^+^ cells derived from recipient mice, our transcriptomics data suggest the presence of recipient c-Kit-derived SPCs and ECs in the aortic grafts. Notably, our flow cytometric analysis of cells from aortic grafts revealed that, although CD31^+^ ECs only represented ~ 2% of total cells, ~ 50% of these ECs were positive for tdTomato (Fig. 4d). This finding provides a strong evidence to support the critical role of c-Kit^+^ cells in EC replacement in the aortic grafts.

We next performed immunostaining of graft sections to analyze the spatial distribution of tdTomato^+^ cells in the aortic grafts. Our results confirmed the presence of tdTomato^+^ (CD31^+^ or CD144^+^) ECs in the grafts, and surprisingly found the cells both in the luminal surface of neointima and inside the grafts (inside the neointima and the adventitia) (Fig. 4e, f). Quantification analyses showed that 25.87 ± 2.11% of CD31^+^ cells were positive for tdTomato in the luminal surface, while 30.15 ± 3.15% of CD31^+^ cells were labelled with tdTomato inside the grafts (Fig. 4g, h). Additional staining of VCAM1 and e-NOS further confirmed these results (Fig. S8d–e). As only recipient mice expressed tdTomato in this model, our single-cell transcriptomics and immunostaining analyses collectively demonstrated that recipient c-Kit^+^ cells serve as EC progenitors and contribute to both luminal EC replacement and microvessel EC repair in transplant arteriosclerosis.

To further address whether c-Kit^+^ cells from donor mice may also participate in EC replacement, tdTomato-labeled aortic segments from tamoxifen-treated Kit-CreER; Rosa26-tdTomato mice were transplanted into BALB/c mice (Fig. S9a). Grafts were harvested 4 weeks later and analyzed. Whole-mount fluorescent microscopy was used to confirm the successful labeling of tdTomato in the donor aorta before transplantation (Fig. S9b). Our results showed that, although the formation of luminal ECs and microvessel ECs were observed, we did not observe any tdTomato^+^ cells in the grafts (Fig. S9c). The disappearance of donor tdTomato^+^ cells may be due to cell apoptosis caused by allo-immune reactions. Taken together, our findings conclude that recipient c-Kit^+^ cells, but not donor c-Kit^+^ cells, are critical for EC replacement in allograft transplantation.

### Nonbone marrow c-Kit^+^ cells replace ECs in transplant arteriosclerosis

**Fig. 5:**
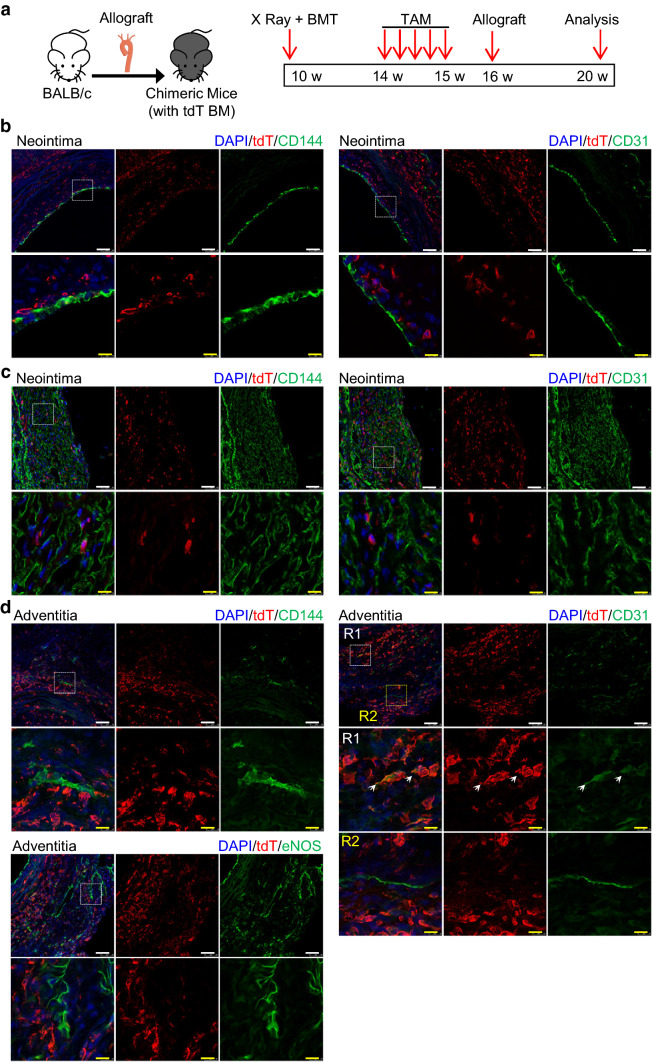
c-Kit-derived ECs in the allograft are not from bone marrow. **a** Chimeric mice were created by transplanting bone marrow from Kit-CreER; Rosa26-tdTomato mice into irradiated C57BL/6J mice. Mice received tamoxifen treatment and allograft transplantation 1 month later. Mice were sacrificed for analysis 1 month after surgery (*n* = 6). **b** Representative images showing luminal ECs in the allograft from chimeric mice, stained with tdTomato, CD31, and CD144. **c** Representative images showing microvessel ECs in the neointimal region of allograft, stained with tdTomato, CD31, and CD144. **d** Representative images showing microvessel ECs in the adventitial region of allograft from chimeric mice, stained with tdTomato, CD31, CD144, and e-NOS. Arrows indicate double-staining cells. Scale bars, 50 μm (white), and 10 μm (yellow). BM(T), bone marrow (transplantation); TAM, tamoxifen; tdT, tdTomato

We next investigated the origin of c-Kit-derived ECs in transplant arteriosclerosis. Chimeric mice were created by transplanting bone marrow cells from Kit-CreER; Rosa26-tdTomato mice to irradiated wild-type C57BL/6J mice, further treated with tamoxifen and subjected to allograft transplantation, and analyzed 4 weeks after surgery (Fig. 5a). Reconstitution of tdTomato^+^ bone marrow cells was confirmed by flow cytometric analysis (Fig. S10). Although tdTomato^+^ cells were detected in the grafts, few CD144^+^ or CD31^+^ ECs on the luminal surface co-expressed tdTomato (Fig. 5b). In agreement with the above finding, most microvessel ECs were negative for tdTomato in both neointima and adventitia (Fig. 5c, d), except that a very small population of CD31^+^ microvessel ECs were observed to co-express tdTomato in the adventitia (Fig. 5d, right panel). These bone marrow-derived tdTomato^+^ cells could be immune cells, as bone marrow-derived c-Kit^+^ cells have been proved to mainly generate immune cells previously [34, 36]. Collectively, these data demonstrate that most luminal and microvessel c-Kit^+^ ECs are derived from nonbone marrow tissues in transplant arteriosclerosis.

We have so far demonstrated a critical role of nonbone marrow c-Kit^+^ cells in EC replacement of transplant arteriosclerosis. However, the exact origin and identity of these c-Kit^+^ cells still remain unclear. We hypothesized that vascular resident c-Kit^+^ cells in the adjacent carotid arteries may be the most possible source. Thus, we further analyzed aortic grafts together with adjacent carotid arteries (Fig. S11a). Immunostaining of the adjacent carotid arteries showed a large number of adventitial tdTomato^+^ cells in the vessels (Fig. S11b), compared to the normal carotid artery (Fig. 2d, right panel), suggesting a possibility of cell expansion of adventitial tdTomato^+^ cells, or cell infiltration of tdTomato^+^ cells from bone marrow or other tissues. Notably, few tdTomato expression was detected in the CD31^+^ endothelial layers of these adjacent carotid arteries. Tissues from the suture sites, which consist of carotid artery, cuff, and aortic graft, were also stained and showed a similar accumulation of tdTomato^+^ cells in the carotid artery (Fig. S11c). While no luminal CD31^+^ ECs co-expressed tdTomato, we did observe some tdTomato^+^CD31^+^ ECs in the medial or adventitial layers of carotid artery or graft regions. We supposed that these cells might be ECs differentiated from vascular resident SPCs (non-ECs), or migrating ECs from the luminal endothelial layers or microvessels. Collectively, these data suggest adjacent vascular c-Kit^+^ cells, including c-Kit^+^ SPCs and a rare population of tdTomato^+^ ECs, as a possible source of ECs in transplant arteriosclerosis.

### c-Kit^+^ SPCs display EC-differentiation potential

**Fig. 6:**
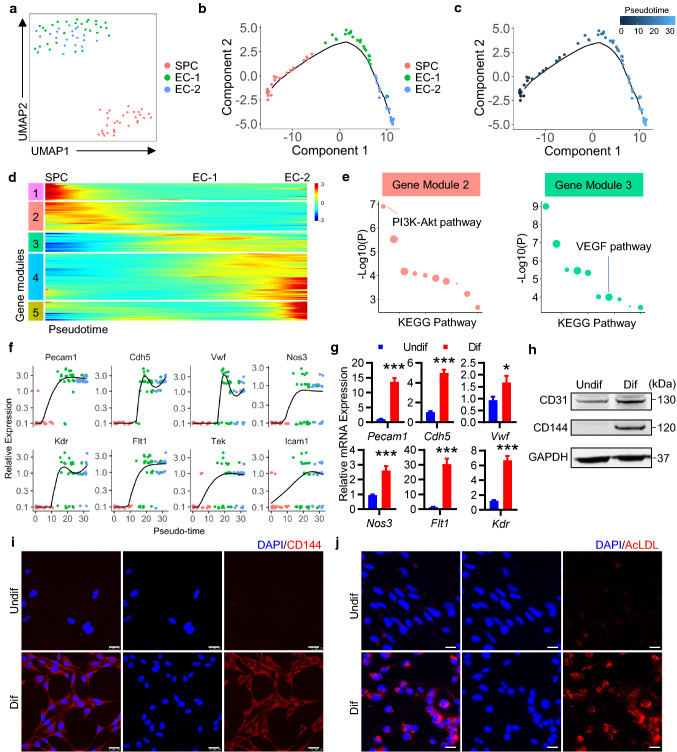
c-Kit^+^ SPCs differentiate into ECs. **a** UMAP plot showing color-coded nonimmune cell clusters from scRNA-seq analysis of tdTomato^+^ cells pooled from aortic grafts by high-resolution clustering. *n* = 79 cells. **b**, **c** Pseudotime trajectory analysis of tdTomato^+^ nonimmune cells (SPC and ECs). Cells are color-coded by cell clusters (**b**) or pseudotime (**c**). **d** Heatmap performed on pseudotime showing expression of gene modules (differentially expressed genes) in all cell clusters. **e** Gene set enrichment analysis of KEGG pathway in gene module 2 and 3. Dot size indicates proportional to the fold enrichment. **f** Kinetics plots showing expression of selected EC marker genes over pseudotime. **g**–**j,** Isolated vascular c-Kit^+^ SPCs were cultured in EGM2 medium for 3 days to induce cell differentiation. **g** Quantitative PCR analyses showing relative gene expression of EC markers normalized to *Gapdh* (*n* = 10 per group). **h** Representative western blot showing expression of endothelial marker CD31 and CD144 (*n* = 5). **i** Representative images showing staining of CD144 in undifferentiated and differentiated c-Kit^+^ SPCs (*n* = 3). **j** Representative images showing uptake of AcLDL in undifferentiated and differentiated cells (*n* = 3). Scale bars, 25 μm (**i**, **j**). Data represent mean ± SEM, *P < 0.05, ***P < 0.001, unpaired two-tailed *t* test (**g**). Undif, undifferentiated cells; Dif, differentiated cells; AcLDL, acetylated low-density lipoprotein

Our above data suggest a possible role of vascular resident c-Kit^+^ cells in EC turnover and replacement. Based on our scRNA-seq data, we also observed a close distribution of tdTomato-labeled c-Kit^+^ SPC and EC populations in Uniform Manifold Approximation and Projection (UMAP) plot (Fig. S8b). High-resolution focused analyses of cell clusters further identified two distinct EC populations (EC-1, EC-2) and one SPC population (Fig. 6a). The close distribution of these populations may indicate a possible cellular transition process based on gene expression patterns. Therefore, we used Monocle [28], an unsupervised algorithm to order cells by progress through cellular biological processes such as cell differentiation, to study the potential of cell differentiation and the dynamic changes of cell expression profiles in these clusters. Results showed that, ordering of these cells in pseudotime arranged all cells into a single trajectory, with SPC occupying the left half of the trajectory, EC-1 in the middle part, and EC-2 on the right half (Fig. 6b, c), suggesting a potential process of c-Kit^+^ SPC differentiation into EC-1 and EC-2. Further analysis identified pseudotime-dependent genes and arranges them into five gene modules (Fig. 6d). Gene set enrichment analysis suggested that gene module 2 was enriched in genes related to PI3K-AKT pathway, while gene module 3 was closely associated with vascular endothelial growth factor (VEGF) pathways (Fig. 6e). During c-Kit^+^ SPC differentiation process, endothelial gene markers were markedly increased over pseudotime, although the expression of some endothelial genes was variable between EC clusters (Fig. 6f). These data collectively suggest a possible differentiation process from c-Kit^+^ SPC to EC in the aortic grafts.

To further confirm this idea, we isolated vascular c-Kit^+^ SPCs from the mouse aorta as previously described [2]. Phenotypic analysis by flow cytometry showed that most isolated c-Kit^+^ cells expressed classical stem/progenitor markers including Sca-1 and CD34, while some also expressed fibroblast or mesenchymal marker CD140a (PDGFRa) and CD105, but not leucocyte marker CD45, endothelial marker CD31 and smooth muscle marker α-SMA (Fig. S12). It conforms that the stem/progenitor properties of these c-Kit^+^ cells are a phenotype similar to the cell cluster of SPC which we identified in our scRNA-seq data. c-Kit^+^ SPCs were then cultured in EC-differentiation medium to test EC-differentiation potential in vitro. After 3-day culture, we observed a significant increase in endothelial-specific genes (*Pecam1*, *Cdh5*, *Kdr*, *Nos3*, *Flt1,* and *Vwf*) in these cells (Fig. 6g), consistent with our pseudotime analysis. Western blot and immunofluorescence analysis also showed an increase in the protein levels of endothelial marker CD31 and CD144 (Fig. 6h-i). Furthermore, these differentiated cells also exhibited the ability to intake acetylated low-density lipoprotein (Fig. 6j), suggesting a functional property of these differentiated cells. Taken together, these data demonstrate the endothelial differentiation potential of vascular c-Kit^+^ SPCs.

### Metabolic reprogramming during c-Kit^+^ SPC differentiation

**Fig. 7:**
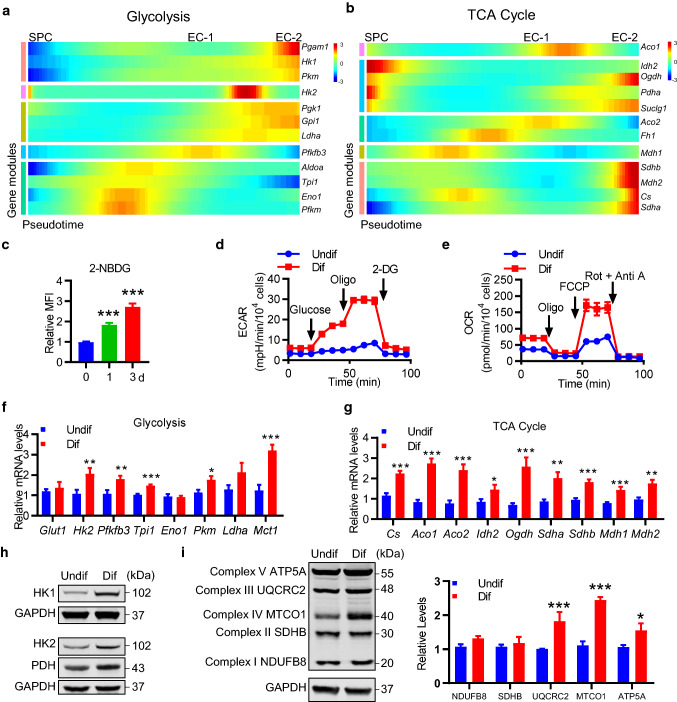
Metabolic reprogramming during c-Kit^+^ SPC differentiation into ECs. **a**, **b** Heatmap showing expression of selected genes related to glycolysis (**a**) and TCA cycle (**b**) over pseudotime. **c–i** c-Kit^+^ SPCs were cultured in EGM2 medium for 3 days or indicated times to induce cell differentiation. **c** Representative histogram and quantification of 2-NBDG MFI showing glucose uptake by flow cytometry (*n* = 4 per group). **d** The ECAR over time was measured at basal level and after the injection of glucose, Oligo, and 2-DG (*n* = 6 in Undif group, *n* = 7 in Dif group). **e** The OCR over time was measured at basal level and after the injection of Oligo, FCCP, Rot, and Anti A (*n* = 3 in Undif group, *n* = 4 in Dif group). **f, g** Quantitative PCR analyses showing relative expression of genes related to glycolysis (**f**) and TCA cycle (**g**) normalized to *Gapdh*. *n* = 10 per group. **h, i** Representative western blot showing expression of glycolytic enzymes (**h**) and mitochondrial respiratory chain complex (**i**) from three independent experiments. Quantification of mitochondrial respiratory chain complex expression is shown (*n* = 3 per group). Data shown are mean ± SEM. *P < 0.05, **P < 0.01, ***P < 0.001, by one-way ANOVA with Dunnett’s test (**c**), or unpaired two-tailed *t* test (**f**, **g**, **i**). Undif, undifferentiated cells; Dif, differentiated cells; Oligo, oligomycin; Rot, rotenone; Anti A, antimycin A

We further investigated possible mechanisms regulating cell differentiation. Previous studies have demonstrated that cellular metabolism, especially glucose metabolism, is critical for EC homeostasis and angiogenesis [40]. Therefore, we sought to test whether cellular metabolism may regulate c-Kit^+^ SPC differentiation. We analyzed the gene expression of metabolic genes over tdTomato-labeled c-Kit^+^ SPC differentiation process based on single-cell transcriptomics data. Our results revealed that, while most genes related to glycolysis showed an increasing trend during tdTomato-labeled c-Kit^+^ SPC differentiation, the levels of genes related to tricarboxylic acid (TCA) cycle were more variable, although increased expression was observed in some genes (Fig. 7a, b). These results from scRNA-seq suggested a possible metabolic reprogramming during c-Kit^+^ SPC differentiation. We further performed in vitro cell experiments to test this idea, using isolated vascular c-Kit^+^ SPCs as described above. Interestingly, similar results were obtained, as evidenced that vascular c-Kit^+^ SPCs exhibited an increased ability to uptake glucose overtime during differentiation by flow cytometric analysis (Fig. 7c). We also used Seahorse extracellular flux assay to perform a detailed analysis of cellular metabolism in vitro, showing a significant increase in both extracellular acidification rate (ECAR), and oxygen consumption rate (OCR) in differentiated cells (Fig. 7d, e). ECAR is an indicator for glycolysis, while OCR indicates mitochondrial oxidative phosphorylation. Quantification of these data revealed a significant increase in parameters of glycolytic flux and mitochondrial oxidative phosphorylation (Fig. S13), suggesting the activation of glycolytic and mitochondrial metabolism in these cells. We further examined the expression of genes related to glycolytic and mitochondrial metabolism. And consistent with the above data and pseudotime analysis, qPCR analyses revealed that a number of genes related to glycolysis and mitochondrial TCA cycle were significantly increased during cell differentiation (Fig. 7f, g). Moreover, Western blot also showed an increase in the expression of several key glycolytic enzymes and mitochondrial proteins (Fig. 7h, i). Taken together, these results provide evidence that c-Kit^+^ SPCs undergo metabolic reprograming (with activation of glycolytic and oxidative metabolism) during differentiation into ECs.

### AKT/mTOR-dependent glucose metabolism regulates endothelial gene expression

**Fig. 8:**
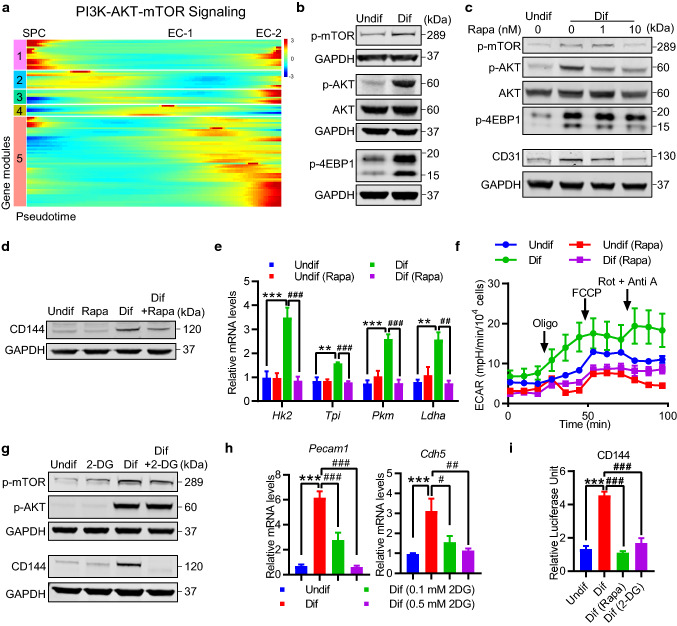
AKT/mTOR-dependent glycolysis regulates endothelial gene expression during c-Kit^+^ SPC differentiation. **a** Heatmap showing expression of hallmark genes related to activation of PI3K-AKT-mTOR signaling over pseudotime. **b–i** c-Kit^+^ SPCs were cultured in EGM2 medium for 3 days or indicated times to induce cell differentiation. **b** Representative western blot analysis showing activation of AKT-mTOR signalling pathways (*n* = 3). **c**, **d** Representative western blot analysis of AKT/mTOR pathways and endothelial markers CD31 (**c**), and CD144 (**d**) in c-Kit^+^ SPCs cultured in EGM2 medium with or without rapamycin (10 nM or indicated doses) for 3 days (*n* = 3). **e** Quantitative PCR analyses showing changes of glycolytic genes in c-Kit^+^ SPCs cultured in EGM2 medium with or without 10 nM rapamycin (*n* = 3 per group). **f** The ECAR over time was measured in c-Kit^+^ SPCs cultured in EGM2 medium with or without 10 nM rapamycin (*n* = 3 per group). **g** Representative Western blot analysis of AKT/mTOR pathways, and CD144 in c-Kit^+^ SPCs cultured in EGM2 medium with or without 2-DG (0.5 mM) for 3 days (*n* = 3). **h** Quantitative PCR analyses showing changes of endothelial markers *Pecam1* and *Cdh5* in c-Kit^+^ SPCs cultured in EGM2 medium with or without indicated doses of 2DG (*n* = 8 per group). **i** Activation of CD144 as determined by a luciferase promoter assay. c-Kit^+^ SPCs cultured in EGM2 medium with or without rapamycin (10 nM), 2-DG (0.5 mM) for 3 days (*n* = 8 per group). Data shown are mean ± SEM. **P < 0.01, ***P < 0.001, ^#^*P* < 0.05, ^##^*P* < 0.01, ^###^*P* < 0.001, by one-way ANOVA with Tukey’s test (**e, h, i**). Undif, undifferentiated cells; Dif, differentiated cells; Rapa, rapamycin

Our pseudotime analysis from scRNA-seq data of tdTomato-labeled c-Kit^+^ cells suggest a possible role of PI3K-AKT pathway during early phase of c-Kit^+^ SPC differentiation into EC (Fig. 6d, e). Previous studies have also suggested that AKT/mTOR signaling pathway is critical in EC differentiation and angiogenesis [41], as well as modulating cellular metabolism [40]. To assess the activation state of this pathway, we further ordered a gene set of hallmark genes up-regulated by activation of the PI3K/AKT/mTOR pathway over pseudotime, and further uncovered five gene modules (Fig. 8a). Despite genes in module 1 were shared by both SPC and EC-2 populations, a large number of genes associated with PI3K/AKT/mTOR pathway were significantly increased during c-Kit^+^ SPC differentiation process, which indicates the activation state of this pathway.

To further confirm the activation state and essential role of AKT/mTOR pathway in this process, we next investigated whether AKT/mTOR pathway may modulate cellular metabolism as well as vascular c-Kit^+^ SPC differentiation in vitro, using isolated vascular c-Kit^+^ SPCs described above. Activation of AKT/mTOR pathway was detected upon cell differentiation (Fig. 8b). Rapamycin, an inhibitor of AKT/mTOR pathway, effectively abrogated the increases in the expression of endothelial marker CD31 and CD144 (Fig. 8c, d), suggesting an essential role of AKT/mTOR pathway in EC differentiation. Of note, while expression of some glycolytic genes was significantly diminished, ECAR, the indicator of glycolysis, was also downregulated by rapamycin (Fig. 8e, f). These results suggest that AKT/mTOR pathway is essential for glycolysis and endothelial gene expression during SPC differentiation. We next tested the necessity of glycolysis in c-Kit^+^ SPC differentiation. 2-Deoxy-D-glucose (2DG), a glucose analog acting as a glycolytic inhibitor, abrogated the increased expression of CD144 proteins during cell differentiation (Fig. 8g). It should be noted that the activation of AKT-mTOR pathway was not prevented by 2DG (Fig. 8g), suggesting that AKT–mTOR pathway serves as upstream of glycolysis. Moreover, gene expression of endothelial markers *Pecam1* and *Cdh5* was also prevented by 2DG (Fig. 8h). While differentiated cells displayed a higher promoter activity of CD144, both rapamycin and 2-DG effectively reversed this increase (Fig. 8i). These data indicate an important role of AKT–mTOR pathway and glycolysis in regulating endothelial gene expression, which may further regulate the protein expression. Taken together, our findings established a possible AKT/mTOR-glycolysis axis in regulating endothelial gene expression during c-Kit^+^ SPC differentiation into EC.

## Discussion

The self-renewal property and differentiation potential of stem cells (including tissue-resident stem cells) make it a powerful source or potential target for cell and gene therapy in clinical application [42]. In the present study, using scRNA-seq and lineage tracing techniques, we uncovered the heterogeneity of vascular SPC, especially c-Kit^+^ cells in the large vessels, and provide the first evidence that endogenous c-Kit^+^ cells contribute to endothelial repair under both physiological and pathological conditions in large vessels.

A recent report [34] has shown the absence of c-Kit expression in intimal ECs of both carotid artery and femoral artery. Interestingly, although we only observed a rare population of intimal ECs in the aorta shortly after tdTomato labeling, a significant increase of tdTomato-labeled c-Kit^+^ ECs was detected in the atheroprone regions, but not athero-resistant regions, during endothelial turnover. These data support the existence of c-Kit^+^ endothelial progenitors, and suggest a clonal expansion of existing c-Kit^+^ ECs or cell differentiation from c-Kit^+^ non-ECs in the aorta, possibly due to the increased endothelial injury and turnover in the atheroprone regions [33], which may explain the different observations in the aorta and carotid/femoral arteries. In a transplant arteriosclerosis model, we also identified c-Kit-derived ECs in the aortic grafts by both scRNA-seq and immunostaining, further supporting the notion that c-Kit^+^ cells serve as endothelial progenitors during homeostasis and vascular remodeling in large vessels. This conclusion seems to contradict the previous report [34], showing that c-Kit^+^ cells minimally contribute to EC repair in femoral artery injury and carotid artery ligation models. We supposed that these contradictory results may be due to different vascular disease models (including different severity of injury and microenvironments). In mild vessel injury conditions (femoral artery injury and carotid artery ligation), intimal ECs might be completely repaired by c-Kit^−^ cells (including c-Kit^−^ ECs or SPCs), and that c-Kit^+^ cells with EC proliferative or differential potential remain quiescent in this microenvironment. While in our severe vessel injury conditions, c-Kit^−^ cells may not be able to repair the whole vessels, or that some specific cytokines, including stem cell factor [36], may be released to activate c-Kit^+^ cells to replace ECs in this condition. Thus, these studies support the notion that differences in the disease models, the extent, and severity of vascular injury may result in different responses of vascular SPCs [35].

In the transplant arteriosclerosis model, c-Kit^+^ cells give rise to both luminal and microvessel ECs. This observation could be interesting, as these two populations of ECs have been shown to exert distinct functions. While luminal ECs maintain vessel integrity and protect against neointima formation [43], arterial microvessel (also known as vasa vasorum) is shown to supply nutrients and oxygen to outer layer of large vessels under homeostasis [44]. Under diseased conditions, expansion of microvessels in both the neointima and adventitia is associated with atherosclerotic plaque growth and progression [44]. Thus, it would also be interesting to distinguish and characterize the luminal and microvessel c-Kit-derived ECs. Of special interest, our scRNA-seq analyses of tdTomato^+^ cells from the aortic grafts also uncovered two distinct subpopulations of ECs. Further combined analysis of these data with immunostaining may help to better distinguish the c-Kit-derived EC populations in the aortic grafts. This information may finally help to develop the therapeutic approach aimed at regression of the microvessels and restoration to the normal architecture of blood vessel.

Although our results suggest a role of c-Kit^+^ cells in aortic EC replacement of the transplant arteriosclerosis model, it should be noted that c-Kit^+^ cells only generated part of these ECs (~ 50% of total ECs from FACS data, ~ 20–30% of total ECs in the aortic grafts from immunostaining). Other c-Kit^−^ cells, including pre-existing ECs, which have recently been shown to participate in the endothelial regeneration or replacement of aorta in a denudation injury model [25], may also contribute to endothelial regeneration or replacement in our mouse models. Indeed, recent lineage tracing studies have also suggested a role of several vascular endothelial progenitor cells (including CD31^hi^Emcn^hi^ cell [45], Procr^+^ cell [46], CD157^+^ cell [47], and CD34^+^CD31^lo^VEGFR2^−^ endovascular progenitor [48]), some of which have also been detected in our scRNA-seq data, in angiogenesis or endothelial regeneration, consistent with previous findings that EC is a heterogeneous population and harbor a stem/progenitor population [4, 49, 50]. These previously identified vascular progenitor cells might also play a role during endothelial turnover and transplant arteriosclerosis.

Our data suggest a nonbone marrow origin of c-Kit-derived ECs in both endothelial turnover and transplant arteriosclerosis models. Although a very small population of bone marrow c-Kit-derived cells was observed to co-express CD31 in aortic grafts, this population did not express CD144 or e-NOS. Thus, we supposed that this population might be immune cells, as CD31 is also expressed by a group of immune cells including neutrophils, monocytes, NK cells, and some T cells [51]. Indeed, our previous study has provided evidence that most bone marrow c-Kit^+^ cells give rise to CD45^+^ leucocytes in this model [36]. These results are consistent with a previous study showing the nonbone marrow origin of neovascular ECs [52]. Despite clear evidence for the nonbone marrow origin of c-Kit-derived aortic ECs, the exact origin still remains unclear. Our data supposed that vascular c-Kit^+^ SPCs in the adjacent vessels may be a possible source, as shown by the detection of adventitial tdTomato^+^ ECs in adjacent carotid artery. This is also supported by a previous study showing that ECs in allograft vasculopathy are mainly migrated from the neighboring vessels [53]. However, further study, which may involve the use of parabiosis model [54] to distinguish local and distal cell populations, is still needed to clearly clarify the source of these c-Kit^+^ cells.

Previous studies have reported the elevation of glucose metabolism in atherosclerotic as well as neointimal lesions [55, 56], which is also associated with high-risk atherosclerotic plaques [57]. In addition, glucose metabolism has been shown to regulate stem cell differentiation [58] and angiogenesis [40]. Both our pseudotime analysis of tdTomato^+^ cells and in vitro study demonstrated a critical role of glycolysis in regulating SPC differentiation into ECs, which is consistent with a report showing that glycolytic switch is required for the trans-differentiation from fibroblast into ECs [59] and supports the importance of glycolytic metabolism in endothelial repair [60]. An AKT/mTOR-dependent glycolysis axis was further suggested to regulate this differentiation process, suggesting a possible role of glycolytic metabolism in EC repair of transplant arteriosclerosis, which may further serve as a potential target in vascular diseases.

In this study, we also showed expression of several previously identified vascular stem cell markers in scRNA-seq data. In additional to the detection of a well-established population of adventitial SPCs, which expressed most of the vascular stem cell markers, several markers were also identified in other cell populations. These results may suggest that vascular stem cells are a heterogeneous population, consisting of a large and specific population of adventitial SPCs, as well as many small subpopulations (with stem cell properties) of certain cell types. Thus, a single marker may not be specific enough to label certain vascular stem cells, which could also be a limitation of our current study. Besides, despite the detection of a c-Kit^+^ SPC population from scRNA-seq data, the small number of this population limited further understanding of the cellular heterogeneity of c-Kit^+^ SPC population. It still remains unclear whether a certain population or the whole population of c-Kit^+^ SPCs has the potential to proliferate and differentiate into ECs, or even other mature cell types. Therefore, the cellular heterogeneity, proliferative, and multilineage potential of endogenous c-Kit^+^ cells require further detailed investigation to understand these heterogeneous populations, including further scRNA-seq analyses of a large number of c-Kit^+^ cells, and using multiple markers [61] to trace different c-Kit^+^ populations with regenerative potential in vivo.

Although a number of studies have suggested a critical role of SPCs in cardiovascular diseases, it remains unclear whether adult stem cell therapy would be beneficial for vascular regeneration in patients [62]. A better understanding of vascular endothelial cell turnover and replacement, and the cellular heterogeneity of vascular SPCs may provide important translational implications. In this study, we demonstrated a critical role of endogenous c-Kit^+^ cells in endothelial repair of large vessels, suggesting vascular c-Kit^+^ cells as a possible candidate for regenerative medicine. Besides, the differentiation of c-Kit^+^ SPCs into ECs is regulated by AKT/mTOR-dependent glucose metabolism. This mechanism might also serve as a potential target for modulating stem cell differentiation. Further investigation and understanding of vascular c-Kit^+^ SPCs is needed, which may provide possibilities for the development of therapeutic approach for vascular diseases in the future.

### Electronic supplementary material

Below is the link to the electronic supplementary material.

Supplementary file1 (PDF 7031 kb)

Supplementary file2 (XLSX 473 kb)

Supplementary file3 (XLSX 498 kb)

Supplementary file4 (XLSX 287 kb)

## Abbreviations

2-DG: 2-Deoxyglucose
ECs: Endothelial cells
ECAR: Extracellular acidification rate
MSC: Mesenchymal stromal cell
NK: Natural killer
OCR: Oxygen consumption rate
RBC: Red blood cells
Sca-1/Ly6a: Stem cell antigen-1/lymphocyte antigen 6 complex, locus A
scRNA-seq: Single-cell RNA-sequencing
SMCs: Smooth muscle cells
SPCs: Stem/progenitor cells
TCA cycle: Tricarboxylic acid cycle
tdTomato/tdT: Tandem dimer Tomato
UMAP: Uniform manifold approximation and projection
VEGF: Vascular endothelial growth factor
