# Supplementary material for: Single-cell gene profiling and lineage tracing analyses revealed novel mechanisms of endothelial repair by progenitors
Source: Cell Mol Life Sci. 2020 Mar 13;77(24):5299–320. doi: 10.1007/s00018-020-03480-4 (PMC11104897; doi:10.1007/s00018-020-03480-4)
Supplement: Supplementary file 1 — Supplementary file1 (PDF 7031 kb) [file 18_2020_3480_MOESM1_ESM.pdf]

SUPPLEMENTAL MATERIALS

Single Cell Gene Profiling and Lineage Tracing Analyses Revealed Novel Mechanisms of Endothelial Repair by Progenitors

Jiacheng Deng[1, 2*], Zhichao Ni[2*], Wenduo Gu[2], Qishan Chen[1], Witold Norbert Nowak[2],Ting Chen[1], Shirin Issa Bhaloo[2], Zhongyi Zhang[2], Yanhua Hu[2], Bin Zhou[3], Li Zhang[1] & Qingbo Xu[1,2]

[1]Department of Cardiology, the First Affiliated Hospital, School of Medicine, Zhejiang University,79 Qingchun Road, Hangzhou, 310003, Zhejiang, China;
[2]School of Cardiovascular Medicine and Science, King's College London, BHF Centre, 125Coldharbour Lane, London SE5 9NU, United Kingdom;
[3]State Key Laboratory of Cell Biology, CAS Center for Excellence in Molecular Cell Science, Institute of Biochemistry and Cell Biology, Shanghai Institutes for Biological Sciences, University of Chinese Academy of sciences, Chinese Academic of Sciences, Shanghai 200031, China.

[*]These authors contributed equally to this work.

Address for Correspondence:

Qingbo Xu, MD, PhD
School of Cardiovascular Medicine & Sciences
King's College London BHF Centre
125 Coldharbour Lane
London SE5 9NU, United Kingdom
Tel: (+44) 20 7848-5322
Fax: (+44) 20 7848-5296
Email: qingbo.xu@kcl.ac.uk

Li Zhang, MD, PhD
Department of Cardiology
The First Affiliated Hospital
Zhejiang University
79 Qingchun Road
Hangzhou 310003, Zhejiang, China
Tel: (+86) 571-87236500
Email: li_zhang@zju.edu.cn

Supplementary Methods

Isolation of single cells from the whole aorta and aortic grafts

For isolation of single cells from the whole aorta, 12-week-old male wildtype and ApoE-/- mice (20 mice per group) were transcardially perfused with 5 mL PBS, and aortas were then harvested from the mice. Perivascular adipose tissues and connective tissues were carefully removed from the aortas. Aortic adventitia was then carefully dissected from the medial and intimal layers. The single cell suspensions were then prepared separately from the aortic adventitia and the remaining medial/intimal layers. To obtain single cell suspensions from the aortic adventitia, the adventitia was washed with PBS for three times, cut into fine pieces and incubated with 2 mg/ml collagenase I (Gibco, 17018029) and 2 mg/ml dispase II (Sigma, D4693) in Hank's balanced salt solution containing calcium and magnesium for a total 40 min. The remaining aortic media and endothelium were cut open longitudinally, washed with PBS for three times, and digested with 1 mg/ml papain (Sigma, P4762), 0.5 mg/ml 1,4-Dithioerythritol (Sigma, D8255), 0.156 mg/ml Taurine (Sigma, T8691); 0.25 mg/ml bovine serum albumin (Sigma, A2058) in PBS. The digestion of both the adventitia and media/intimal layers was performed at 37 ℃ with reciprocal shaking (125 rpm). Detached cells were collected, suspended in DMEM with 10% FBS (Gibco, 10270) and the tissues were then incubated with fresh digestion solutions every 10 min. Specifically, the remaining adventitia and media/intimal tissues were cut into fine pieces after 20 min's digestion, and further incubated with corresponding digestion solution for another 20 min. All digested cells from one sample were pooled together at the end of the digestion process. Digested cells were then passed through a 40 μm cell strainer and stained with LIVE/DEAD ™ Fixable Near-IR Dead Cell Stain Kit (Invitrogen, L34975, 1:1000) and Hoechst 33342 (Invitrogen, H3570, 1:1000) for 20 mins. Specifically, digested cells from the medial/intimal layers were first stained with CD31-AF647 (Biolegend,102516, 1:1000) for an additional 20 min. After PBS washing, cells were resuspended in PBS and single nucleated live cells (Hoechst$^+$/Dead Cell Stain$^-$) were sorted into PBS with 0.04% BSA using a BD FACS ARIA II Flow Cytometer (BD Biosciences). For cells from the medial/intimal layers, endothelial cells (CD31$^+$Hoechst$^+$/Dead Cell Stain$^-$) and non-endothelial cells (CD31$^-$ Hoechst$^+$/Dead Cell Stain$^-$) were sorted into PBS with 0.04% BSA. A single cell preparation comprising approximately 50% endothelial cells and 50% non-endothelial cells (mostly smooth muscle cells) was then prepared. For isolation of single tdTomato$^+$ cells from the aortic grafts, a similar protocol was used as describe above. Specifically, aortic grafts from 14 or 16-week-old Kit-CreER; Rosa26-tdTomato mice (n=4 mice) were isolated for scRNA-seq analysis. Following a similar digestion protocol as described above, all digested cells from one sample were pooled together at the end of the digestion process. Digested cells were then passed through a 40 μm cell strainer and stained with DRAQ5 (Abcam, ab108410, 1:1000) and CD31-FITC (Invitrogen, 11-0311-81, 1:1000) for 30 mins. DAPI was added to the cell just before loading to flow cytometer. Single nucleated live tdTomato$^+$ cells (DRAQ$^+$/DAPI$^-$) were sorted into PBS with 0.04% BSA using a BD FACS ARIA II Flow Cytometer (BD Biosciences).

Single-cell RNA-sequencing and data analysis.

For single-cell RNA-sequencing (scRNA-seq) of four pooled samples obtained from the adventitia and the intimal/medial layers of aorta, from wildtype or ApoE-/- mice, a Chromium^{TM} Single Cell 3' Reagent Kit v2 chemistry (10X Genomics) was used and a standard protocol was followed. Library was generated and sequenced on a NovaSeq platform (Illumina) with paired-end 150 bp (PE 150) sequencing strategy. Single cell RNA-sequencing raw data were processed using Cell Ranger (version 2.1.1). Briefly, "Cellranger mkfastq" was used to demultiplex raw data and generate FASTQ files, which were further processed by "Cellranger count" to align reads to the mouse mm10 reference, count the number of barcode and UMI, and to generate feature-barcode matrices. To reduce batch effect, we also used "Cellranger aggr" to aggregate sequencing data from different libraries to the same sequencing depth. Specifically, we have aggregated data from the adventitia and intimal/medial

layers from wildtype or ApoE-/- mice. Data from wildtype and ApoE-/- mice (all four pooled samples) were also aggregated together for further analyses. After aggregation of the samples, Seurat [1] v3 was used for downstream analyses. Analysis were performed with default parameters unless otherwise specified. Briefly, for the analyses of the whole aorta from wildtype and ApoE-/- mice (aggregation data of all four pooled samples), gene features expressed in at least 3 cells and cells with at least 200 detected genes were kept, followed by filtering cells displaying over 3,500 and less than 500 gene features, and cells having over 8% mitochondrial counts. 9,745 cells remained post-filtering and data were normalized by "LogNormalize". 2,000 most highly variable genes were then selected and all genes were scaled by using "ScaleData". Principle component analysis was then performed on selected highly variable genes, and the first 25 principle components with a resolution of 0.2 were used for cell clustering and Uniform Manifold Approximation and Projection (UMAP) [2] visualization. The marker genes upregulated in each cluster were identified by "FindAllMakrers" (min.pct=0.25, logfc.threshold=0.25). Integrating analysis of the whole aorta data from our dataset with three other published datasets[3-6] was also analyzed using Seurat v3. A standard integrated analysis was performed with default parameters. 19,234 cells remained post-filtering. Specifically, 2,000 most highly variable genes were selected for analysis and the first 30 principle components with a resolution of 0.2 was used for cell clustering and UMAP visualization.

For single-cell RNA-sequencing (scRNA-seq) of tdTomato$^+$ cells obtained from aortic grafts, a similar method was used as described above. Specifically, a Chromium^{TM} Single Cell 3' Reagent Kit v3 chemistry (10X Genomics) was used and a standard protocol was followed. Library was generated and sequenced on a Hiseq 2500 System (Illumina). Single cell RNA-sequencing raw data were processed using Cell Ranger (version 3.0.2). Aggregation data was also analysed by Seurat [1] v3 for cell clustering and differentially expressed genes following a standard protocol. Specifically, cells displaying less than 500 gene features, and cells having over 10% mitochondrial counts were filtered. The first 30 principle components with a resolution of 0.2 were used for cell clustering. Focused sub-clustering analyses of nonimmune cells (SPC/EC) were performed and the first 20 principle components with a resolution of 1.1 were used for cell clustering. A contamination of immune cells (2 cells) was also excluded from these populations for subsequent analysis. Pseudotime trajectory analysis was performed using Monocle 2[7]. We used differentially expressed genes acquired from the above Seurat analysis to order cells in pseudotime. For analysis of PI3K-AKT-mTOR signaling over pseduotime, we used a gene set "HALLMARK_PI3K_AKT_MTOR_SIGNALING" from GSEA, which includes genes up-regulated by activation of the PI3K/AKT/mTOR pathway. Human gene names were first converted to mouse gene names using "biomaRt"[8] before analysis. Gene set enrichment analysis of KEGG pathway was analyzed using DAVID[9] bioinformatics resources.

Supplemental References

1. Butler A, Hoffman P, Smibert P, Papalexi E, Satija R (2018) Integrating single-cell transcriptomic data across different conditions, technologies, and species. Nat Biotechnol 36 (5):411-420. doi:10.1038/nbt.4096
2. McInnes L, Healy J, Melville J (2018) UMAP: Uniform Manifold Approximation and Projection for Dimension Reduction. arXiv e-prints
3. Dobnikar L, Taylor AL, Chappell J, Oldach P, Harman JL, Oerton E, Dzierzak E, Bennett MR, Spivakov M, Jorgensen HF (2018) Disease-relevant transcriptional signatures identified in individual smooth muscle cells from healthy mouse vessels. Nat Commun 9 (1):4567. doi:10.1038/s41467-018-06891-x
4. McDonald AI, Shirali AS, Aragon R, Ma F, Hernandez G, Vaughn DA, Mack JJ, Lim TY, Sunshine H, Zhao P, Kalinichenko V, Hai T, Pelegrini M, Ardehali R, Iruela-Arispe ML (2018) Endothelial Regeneration of Large Vessels Is a Biphasic Process Driven by Local Cells with Distinct Proliferative Capacities. Cell Stem Cell 23 (2):210-225 e216. doi:10.1016/j.stem.2018.07.011
5. Tabula Muris C, Overall c, Logistical c, Organ c, processing, Library p, sequencing, Computational data a, Cell type a, Writing g, Supplemental text writing g, Principal i (2018) Single-cell transcriptomics of 20 mouse organs creates a Tabula Muris. Nature 562 (7727):367-372. doi:10.1038/s41586-018-0590-4
6. Kalluri AS, Vellarikkal SK, Edelman ER, Nguyen L, Subramanian A, Ellinor PT, Regev A, Kathiresan S, Gupta RM (2019) Single Cell Analysis of the Normal Mouse Aorta Reveals Functionally Distinct Endothelial Cell Populations. Circulation. doi:10.1161/CIRCULATIONAHA.118.038362
7. Qiu X, Mao Q, Tang Y, Wang L, Chawla R, Pliner HA, Trapnell C (2017) Reversed graph embedding resolves complex single-cell trajectories. Nat Methods 14 (10):979-982. doi:10.1038/nmeth.4402
8. Durinck S, Spellman PT, Birney E, Huber W (2009) Mapping identifiers for the integration of genomic datasets with the R/Bioconductor package biomaRt. Nat Protoc 4 (8):1184-1191. doi:10.1038/nprot.2009.97
9. Huang da W, Sherman BT, Lempicki RA (2009) Systematic and integrative analysis of large gene lists using DAVID bioinformatics resources. Nat Protoc 4 (1):44-57. doi:10.1038/nprot.2008.211

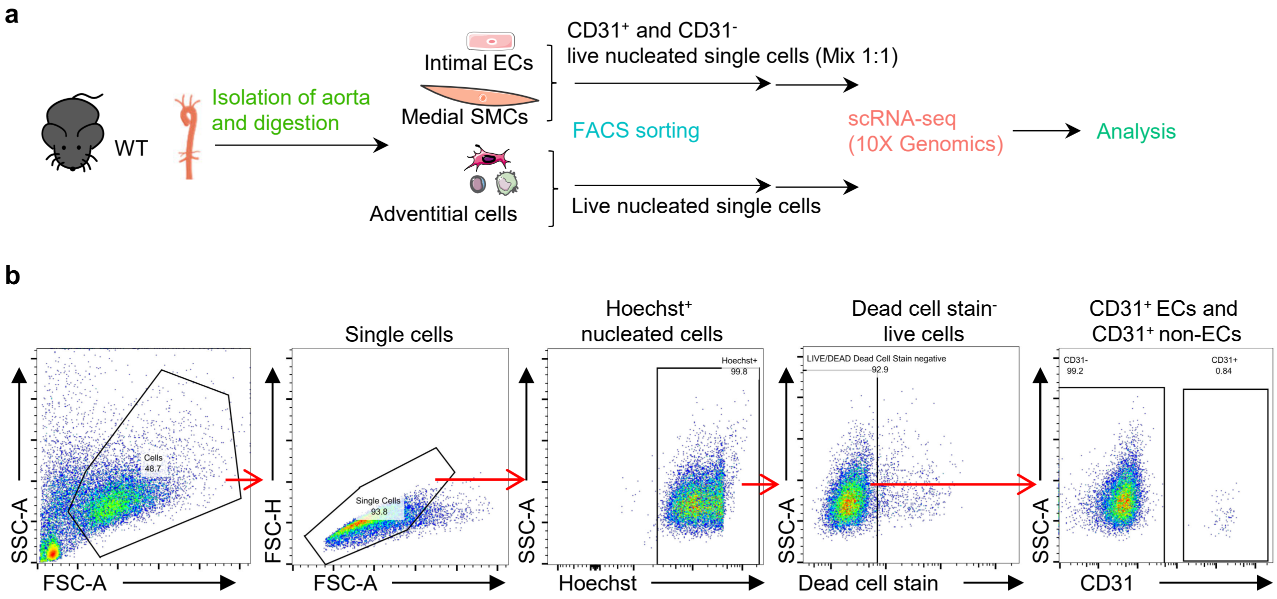

Fig. S1 Isolation of live nucleated single cells from the aortas of wildtype and ApoE-/- mice for scRNA-seq analysis. **a** Schematic showing experimental procedure for preparation of live nucleated single cells from aortas for scRNA-seq analysis. Single cells were separately prepared from the adventitia and intimal/medial layers and subjected to scRNA-seq (n=20 mice per group). Specifically, $CD31^+$ ECs and $CD31^-$ non-ECs were sorted from the intimal/medial layers and mixed at a 1:1 ratio before scRNA-seq. **b** Representative gating strategy of live nucleated ($Hoechst^+$Dead Cell $Stain^-$) single cells preceding gating of $CD31^+$ ECs and $CD31^-$ non-ECs.

a

	Aggregated data of WT mice	Aggregated data of ApoE-/- mice	Aggregated data of WT and ApoE-/- mice
Estimated Number of Cells	5,156	5,466	10,622
Mean Reads per Cell	221,237	184,099	181,034
Median Genes per Cell	1,900	1,519	1,674
Number of Reads	1,140,699,229	1,006,289,465	1,922,948,326
Median UMI Counts per Cell	5,403	4,452	4,826
Fraction Reads in Cells	87.5%	84.8%	86.1%

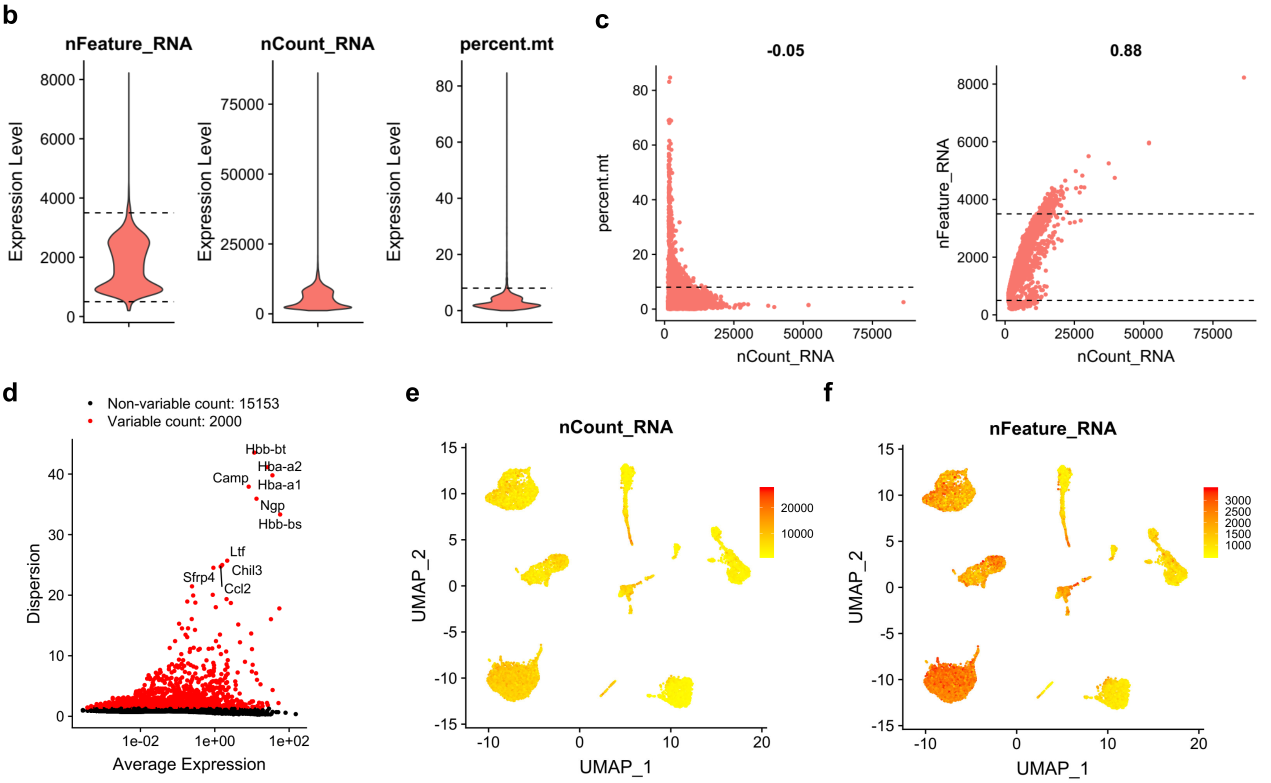

Fig. S2 Quality control data for scRNA-seq of mouse aortic cells. **a** Overview of scRNA-seq data quantity metrics for all aggregated data. **b-f** Quality control analysis for aggregated data of WT and ApoE-/- mice. **b** Violin plots showing expression level of gene features, cell counts (UMI), and mitochondrial percentage in all cells. Dashed lines mark upper or lower cut-offs for filtering cells for downstream analysis. **c** Graphs showing relationship between expression level of gene features, cell counts and mitochondrial. Dashed lines mark upper or lower cut-offs for filtering cells for downstream analysis. **d** Graphs showing relationship between average expression and dispersion. Red dots shown are highly variable genes selected for downstream analysis. **e** UMAP-plot showing color-coded number of counts per cell. **f** UMAP-plot showing color-coded genes per cell.

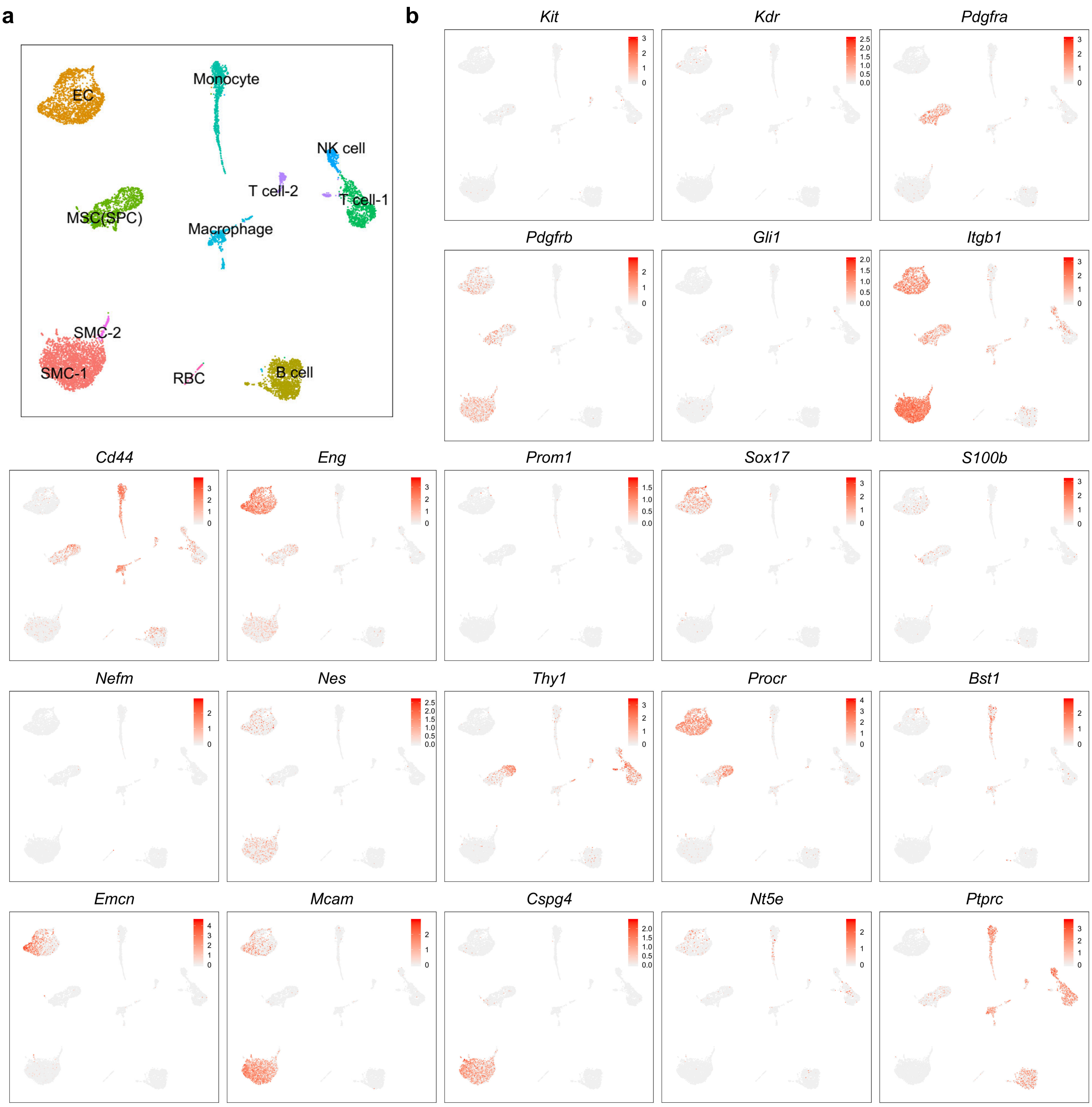

Fig. S3 Related to Fig. 1a-1d. ScRNA-seq analysis of previously reported vascular stem/progenitor cell markers in the whole aorta. **a** UMAP plot showing 11 color-coded cell clusters with putative biological indentities. n=9,745 individual cells. **b** Feature plot showing average expression (color-scaled) of previously reported stem progenitor marker genes in each cell cluster.

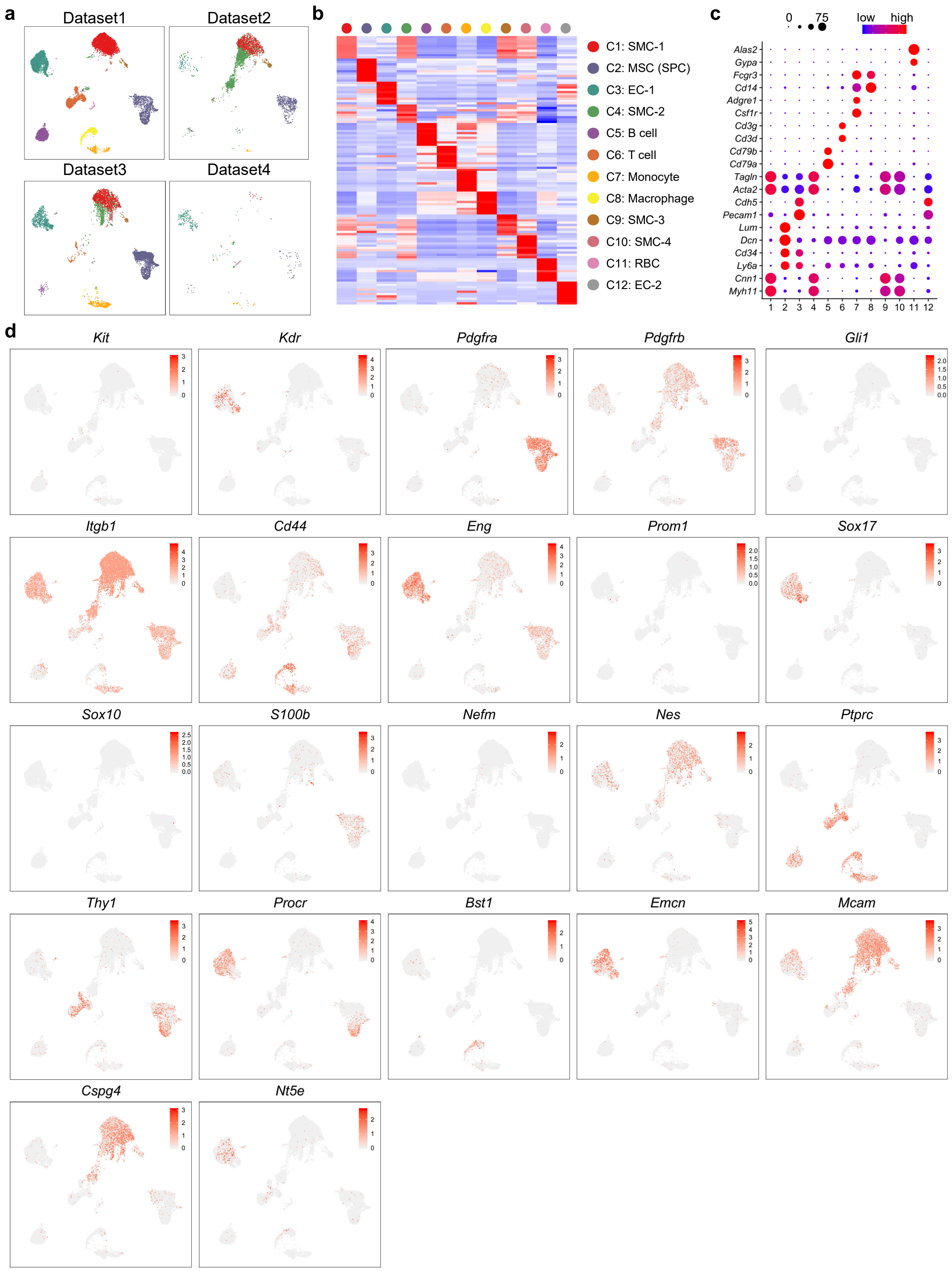

Fig. S4 Related to Fig. 1e-1f. Integrated analysis of the whole aorta from four scRNA-seq datasets. **a** UMAP plot showing color-coded cell clusters across the four scRNA-seq datasets. Putative biological identity of each cell cluster is defined on the right. n=19,234 individual cells. **b** Heatmap showing top 10 differentially expressed genes in each cell cluster. Normalized gene expression is shown. **c** Dot plot showing average expression (color-scaled) of previously reported stem progenitor marker genes in each cell cluster. Dot size reflects the proportion of cells expressing the selected gene. **d** Feature plot showing average expression (color-scaled) of selected marker genes in each cell cluster.

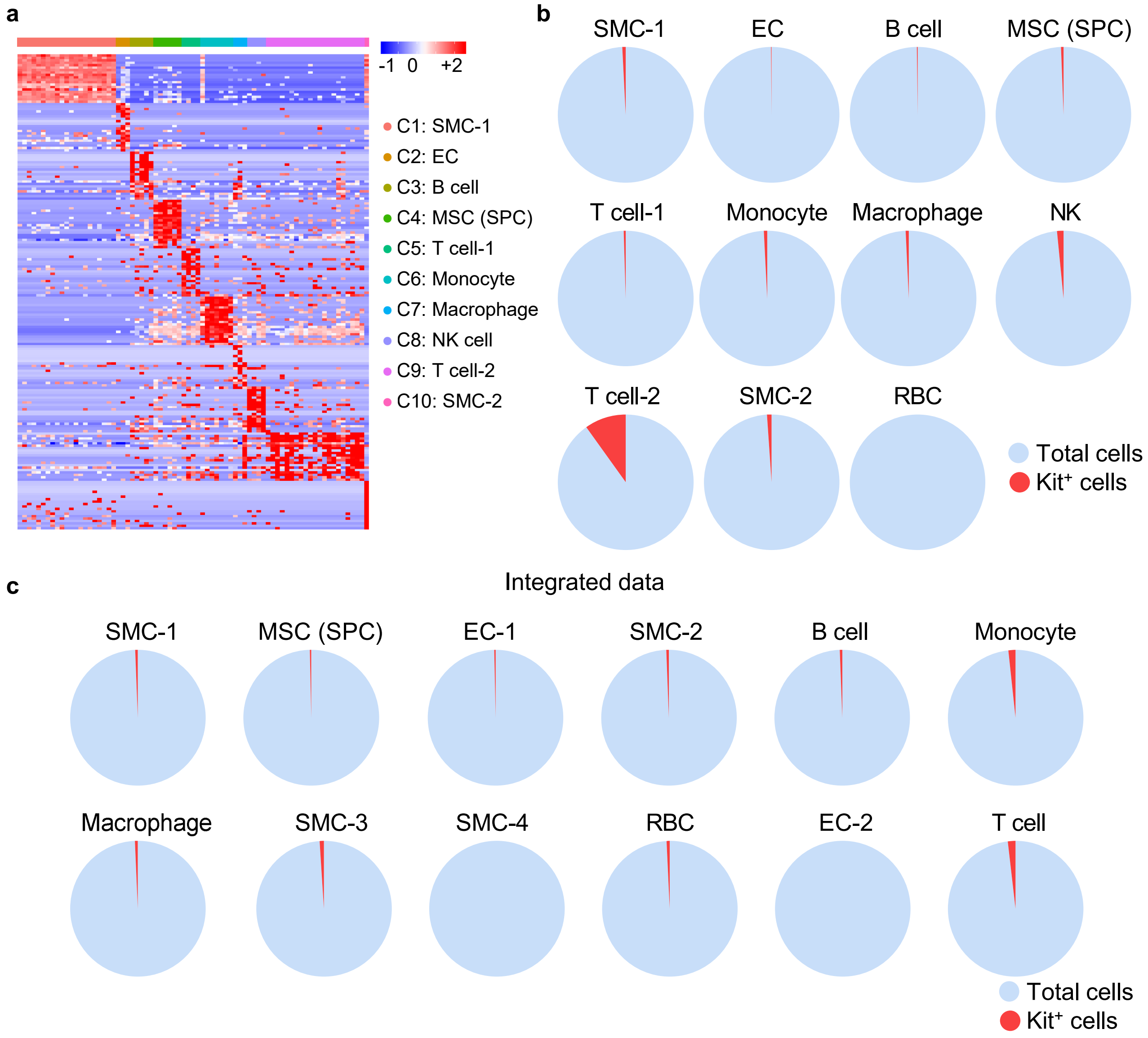

Fig. S5 ScRNA-seq analysis of *Kit*-expressing cells in the whole aorta (aggregated data of WT and ApoE-/- mice). **a** Heatmap showing top 20 differentially expressed genes in each cell cluster (*Kit*-expressing cells are shwon). Putative biological identity of each cell cluster is defined on the right. **b** Graph showing the total cell number of each biological identities and the number of *Kit*-expressing cells in each cluster (single dataset). **c** Graph showing the total cell number of each biological identities and the number of *Kit*-expressing cells in each cluster (four integrated datasets).

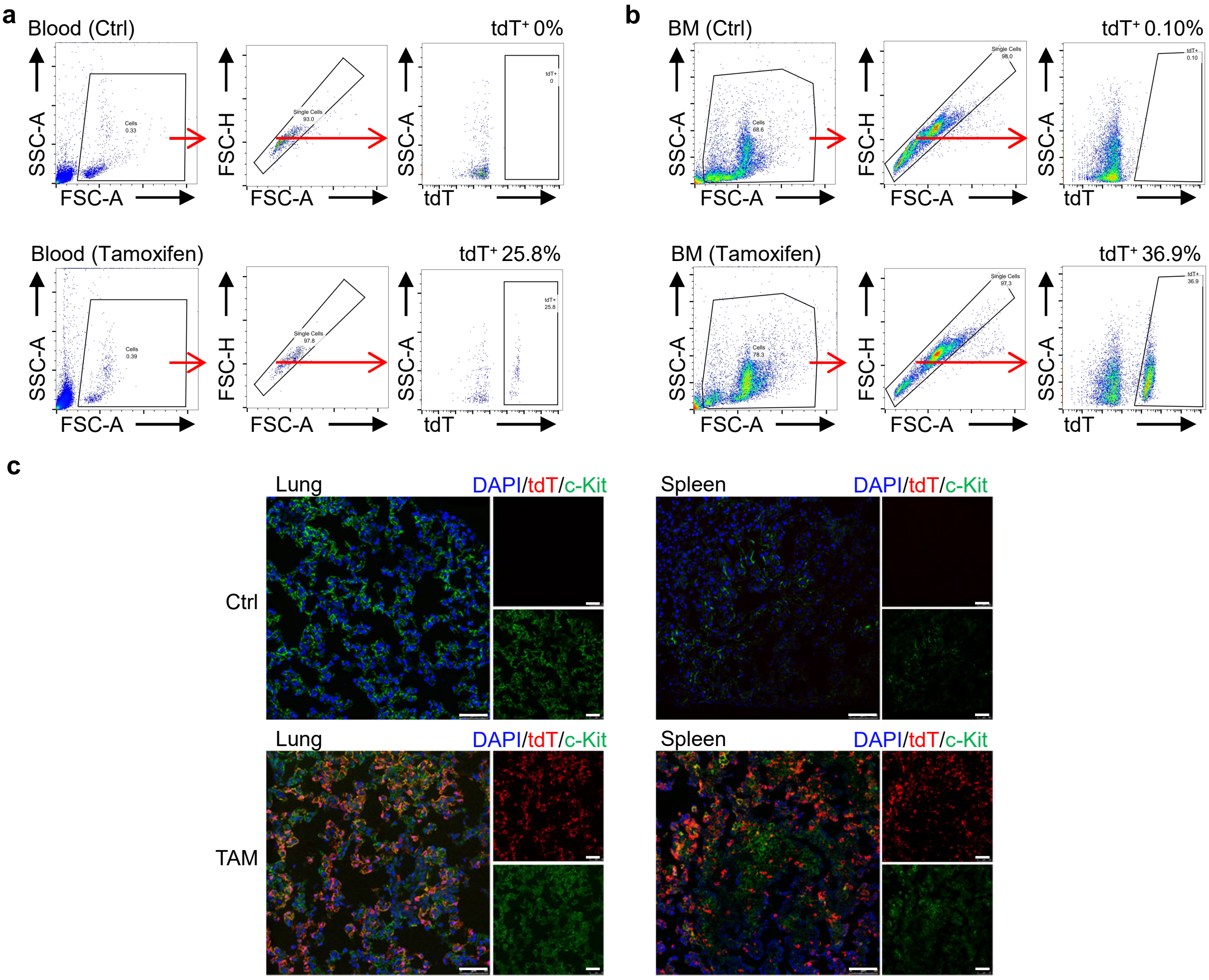

Fig. S6 Related to Fig. 2. Verification of tdTomato labelling in Kit-CreER; Rosa26-tdTomato mice. **a-b** Kit-CreER; Rosa26-tdTomato mice were treated with tamoxifen or control corn oil as described in Figure 2a. Flow cytometric analysis of tdTomato$^+$ cells in blood (**a**) and bone marrow (**b**) (n=3). **c** Representative images of immunostaining showing expression of tdTomato and c-Kit in the lung and spleen. Scale bars, 50 μm. BM indicates bone marrow; Ctrl, corn oil control; TAM, tamoxifen; tdT, tdTomato.

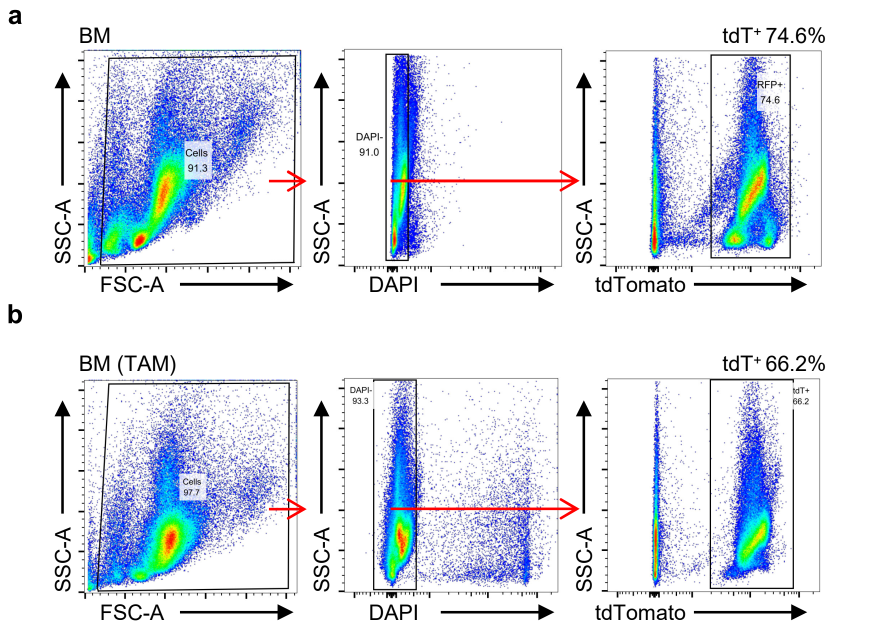

Fig. S7 Related to Fig. 3. Verification of tdTomato labeling in mice. Representative flow cytometric analysis of tdTomato-labelled bone marrow cells collected from Kit-CreER; Rosa26-tdT mice (**a**) as described in Fig. 3a and chimeric mice (**b**) as described in Fig. 3c. BM indicates bone marrow; TAM, tamoxifen; tdT, tdTomato.

a

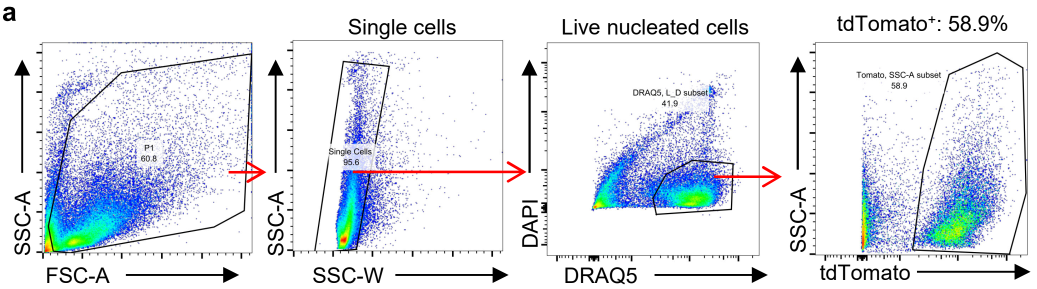

b

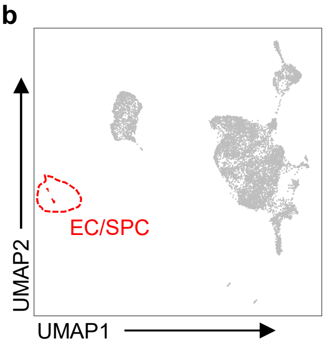

c

Time	Aggregated	2 week	4 week
Total cell num	11,154	5,271	5,883
Total cell num (filtered)	9,618	4,585	5,033
EC/SPC/FB	81	26	55

d, **e**

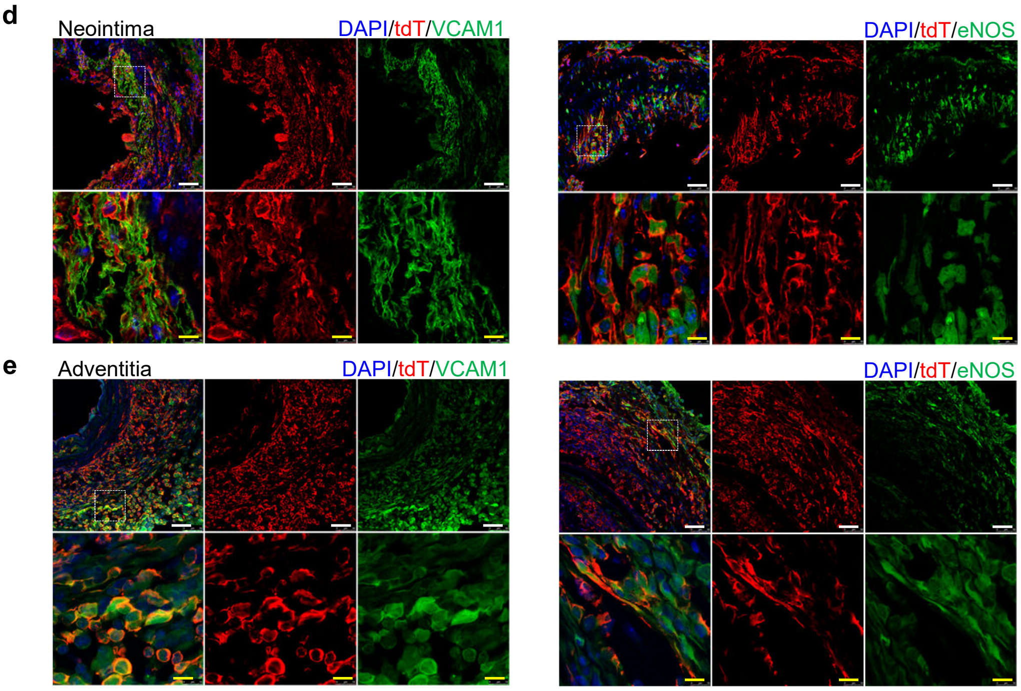

Fig. S8 Related to Fig. 4. Recipient c-Kit⁺ cells repopulate ECs in allograft. **a** Representative gating strategy for sorting live nucleated (DRAQ5⁺DAPI⁻) tdTomato⁺ single cells. **b** UMAP plot showing cell clusters from unbiased clustering analysis of tdTomato⁺ cells pooled from aortic grafts. n=9,618 cells. A mixed population of EC/SPC are labeled (red). **c** Overview of cell numbers for scRNA-seq data. **d** Representative images showing microvessel ECs in the neointimal region of allograft, stained with tdTomato, e-NOS and VCAM-1. **e** Representative images showing microvessel ECs in the adventitial region of allograft, stained with tdTomato, e-NOS and VCAM-1. Scale bars, 50 μm (white) and 10 μm (yellow). tdT, tdTomato.

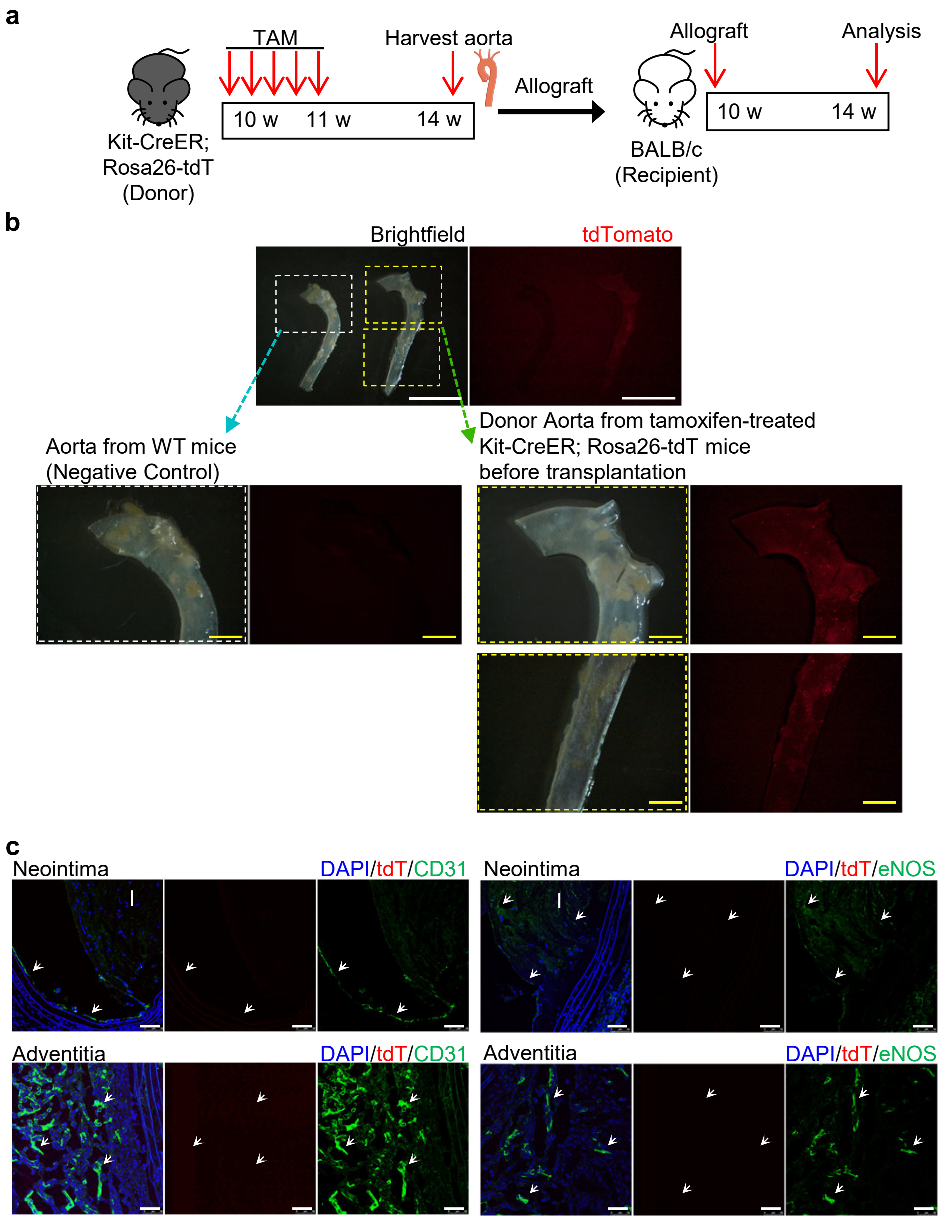

Fig. S9 c-Kit-derived ECs in the allograft are not of donor origin. **a** Kit-CreER; Rosa26-tdTomato mice were pulsed with tamoxifen and aortas were harvested. tdTomato labelling of donor aortas were confirmed by whole-mount fluorescence analysis, then transplanted into recipient BALB/c mice and analysed 4 weeks later (n=6 mice per group). **b** Representative whole-mount images showing labeling of tdTomato in donor aorta harvested from tamoxifen-treated Kit-CreER; Rosa26-tdTomato mice (Aorta shown on the right). Aorta from wildtype mice served as negative control (Aorta shown on the left). Scale bars: 5 mm (white), 1 mm (yellow). **c** Representative images showing neointimal and adventitial regions of aortic grafts stained with tdTomato, CD31 and eNOS**.** Scale bars, 50 μm. TAM indicates tamoxifen; tdT, tdTomato.

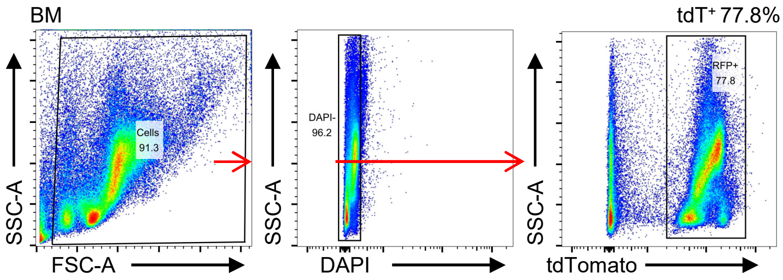

Fig. S10 Related to Fig. 5. Verification of tdTomato labeling in chimeric mice after bone marrow transplantation. Representative flow cytometric analysis of tdTomato-labelled bone marrow cells collected from chimeric mice as described in Fig. 5a. n=6. BM indicates bone marrow; TAM, tamoxifen; tdT, tdTomato.

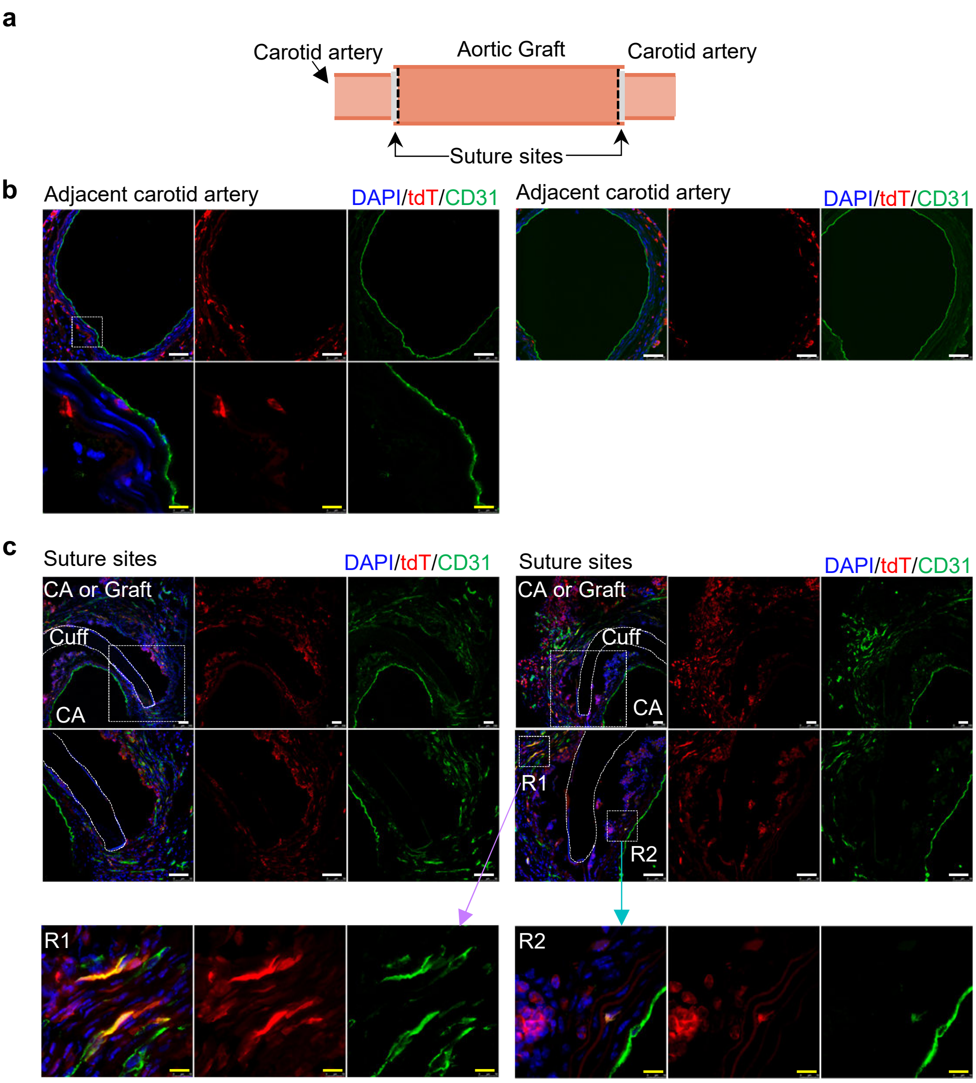

Fig. S11 Related to Fig. 4. Analysis of aortic grafts with adjacent carotid arteries. **a** Aortic graft with adjacent carotid arteries were collected for analysis from mice described in Fig. 4a. Arrows indicate adjacent carotid artery or suture sites. **b** Representative images showing tdTomato^{+} cells and CD31^{+} ECs in the adjacent carotid artery. **c** Representative images showing expression of tdTomato and CD31 in the suture sites, which consist of carotid artery, cuff and outside regions of carotid artery or aortic graft. Scale bars, 50 μm (white) and 10 μm (yellow). tdT indicates tdTomato; CA, carotid artery; R1/2, region 1/2.

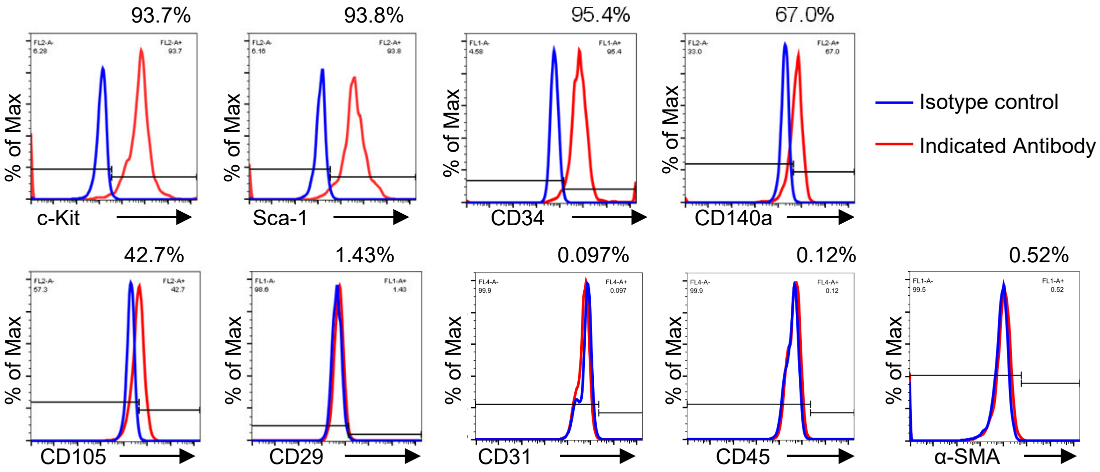

Fig. S12 Phenotyping of vascular c-Kit+ cells isolated from aortic adventitia. Representative histogram showing expression levels of markers in c-Kit+ SPCs (red). Corresponding IgG (blue) was used as isotype controls (n=3).

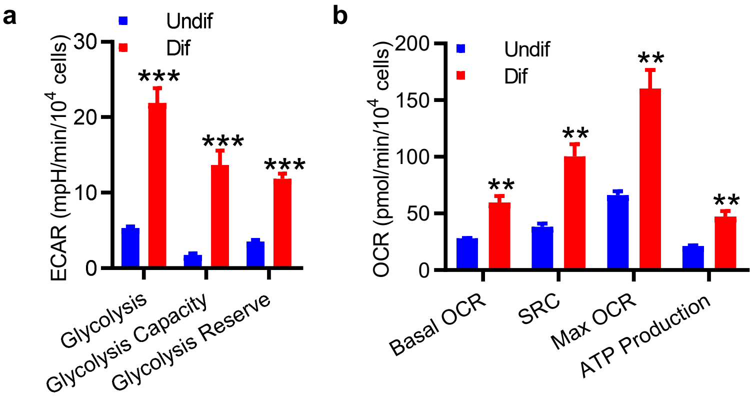

Fig. S13 Related to Fig. 7. Metabolic reprogramming during c-Kit⁺ SPC differentiation. **a** Quantification of glycolysis, glycolysis capacity and glycolysis reserve are shown (n=6 in Undif group, n=7 in Dif group). **b** Quantification of basal OCR, SRC, max OCR, proton leak and ATP production were shown (n=3 in Undif group, n=4 in Dif group). Data shown are mean ± SEM. $^{**}P<0.01$, $^{***}P<0.001$, by unpaired two-tailed t test (**a** and **b**).

Table S1. Related to Fig. 1b. Upregulated differentiated expressed genes in each cluster (Total cells from aggregated data).

Find attached excel file (Supplemental Table S1.xlsx).

Table S2. Related to Fig. 1e and Fig. S4. Upregulated differentiated expressed genes in each cluster (Total cells from integrated data of four scRNA-seq datasets).

Find attached excel file (Supplemental Table S2.xlsx).

Table S3. Related to Fig. 1g and Figure S5a. Upregulated differentiated expressed genes in each cluster (*Kit*-expressing cells from aggregated data).

Find attached excel file (Supplemental Table S3.xlsx).

Table S4. List of Primers for qPCR.

Gene	Forward (5' to 3')	Reverse (5' to 3')
Pecam1	CACCCATCACTTACCACCTTATG	TGTCTCTGGTGGGCTTATCT
Cdh5	AAGAAACCGCTGATCGGCA	TCGGAAGAATTGGCCTCTGTC
Kdr	TGAAATTGAGCTATCTGCCGG	TTTGAAGGTGGAGAGTGCCAG
Nos3	GGCTGGGTTTAGGGCTGTG	CTGAGGGTGTCGTAGGTGATG
Flt1	ACCTCACCGTGCAAGGAACC	ACTGCTCATCCAGGGGAACT
Vwf	CTTCTGTACGCCTCAGCTATG	GCCGTTGTAATTCCCACACAAG
Kit	CCTCCTGCCCTTTATCCTTTAG	GACCTCCAAACCAGCTTACTT
Glut1 (Slc2a1)	GTGACGATCTGAGCTACGGG	AACGGACGCGCTGTAACTAT
Hk2	ATGATCGCCTGCTTATTCACG	CGCCTAGAAATCTCCAGAAGGG
Pfkfb3	ACAGGACACGCCTTCCTTTC	CGCAAAAACCTGAGAAGCGA
Tpi1	ACTGCCCATCCCTCTACCTG	TAATAGGGCGGTGGAAACGG
Eno1	CGTCCACTGGCATCTACG	GAGCAGGCGCAATAGTTTTA
Pkm	ACCCTGGACAACGCTTACAT	ACCAGGAAGTCAGCGCCTTT
Ldha	TGCACTAGCGGTCTCAAAAG	GTCAACAAGGGCAAGCTCATC
Mct1 (Slc16a1)	GTGCAACGACCAGTGAAGTA	ACAACCACCAGCGATCATTA
Cs	TGTTTCAGGGGCCTTTAAGACT	CATGCTTCAGTCCCGGTCAT
Aco1	AGAACCCATTTGCACACCTTG	AGCGTCCGTATCTTGAGTCCT
Aco2	AACCGGCCTCTTACTCTCTCA	CAGGTATGTCTTTCCCCGCTC
Idh2	GGAGAAGCCGGTAGTGGAGAT	GGTCTGGTCACGGTTTGGAA
Ogdh	AGGGCATATCAGATACGAGGG	CTGTGGATGAGATAATGTCAGCG
Sdha	GGCTTTCACTTCTCTGTTGGT	AGAAAGGCCAAATGCAGCTCG
Sdhb	ATTTACCGATGGGACCCAGAC	GTCCGCACTTATTCAGATCCAC
Mdh1	GTTGCCTGACTCGCTTGGA	TTACATCATCAGCGGTTACACC
Mdh2	AAGGTTGTGATGTGGTGGTCA	TAGCGTTGGTGTTGAACAGGT
Etv2	ACCGTCAGAACAAGCATCCA	AAACCAAATTCCGCTCCCA
Gapdh	TCTCCCTCACAATTTCCATCC	GGGTGCAGCGAACTTTATTG
